# Excess crossovers impede faithful meiotic chromosome segregation in *C. elegans*

**DOI:** 10.1371/journal.pgen.1009001

**Published:** 2020-09-04

**Authors:** Jeremy A. Hollis, Marissa L. Glover, Aleesa J. Schlientz, Cori K. Cahoon, Bruce Bowerman, Sarah M. Wignall, Diana E. Libuda

**Affiliations:** 1 Department of Molecular Biosciences, Northwestern University, Evanston, IL, United States of America; 2 Institute of Molecular Biology, Department of Biology, University of Oregon, Eugene, OR, United States of America; University of California, Davis, UNITED STATES

## Abstract

During meiosis, diploid organisms reduce their chromosome number by half to generate haploid gametes. This process depends on the repair of double strand DNA breaks as crossover recombination events between homologous chromosomes, which hold homologs together to ensure their proper segregation to opposite spindle poles during the first meiotic division. Although most organisms are limited in the number of crossovers between homologs by a phenomenon called crossover interference, the consequences of excess interfering crossovers on meiotic chromosome segregation are not well known. Here we show that extra interfering crossovers lead to a range of meiotic defects and we uncover mechanisms that counteract these errors. Using chromosomes that exhibit a high frequency of supernumerary crossovers in *Caenorhabditis elegans*, we find that essential chromosomal structures are mispatterned in the presence of multiple crossovers, subjecting chromosomes to improper spindle forces and leading to defects in metaphase alignment. Additionally, the chromosomes with extra interfering crossovers often exhibited segregation defects in anaphase I, with a high incidence of chromatin bridges that sometimes created a tether between the chromosome and the first polar body. However, these anaphase I bridges were often able to resolve in a LEM-3 nuclease dependent manner, and chromosome tethers that persisted were frequently resolved during Meiosis II by a second mechanism that preferentially segregates the tethered sister chromatid into the polar body. Altogether these findings demonstrate that excess interfering crossovers can severely impact chromosome patterning and segregation, highlighting the importance of limiting the number of recombination events between homologous chromosomes for the proper execution of meiosis.

## Author summary

Meiosis is a process that ensures developing eggs and sperm contain the correct number of chromosomes. Failure to accurately segregate chromosomes during meiosis is one of the leading causes of birth defects and miscarriages. During meiosis, crossover events must form between the homologous chromosomes to ensure proper chromosome segregation. Although crossover events are required for proper chromosome segregation in most organisms, crossover numbers are limited even when the meiotic cell is overloaded with DNA breaks, the initiating events for crossovers. This stringent limitation of crossovers in multiple organisms suggests that there are negative consequences to having too many crossovers, but this has not been formally tested. In this study, we find that increasing crossover number negatively impacts chromosome segregation during meiosis by altering chromosome-associated structures, which leads to misalignment of the chromosomes on the meiotic spindle. Moreover, chromosomes with excess crossovers often have large chromatin bridges during the chromosome segregation process, but we find that these bridges can be corrected by at least two mechanisms. Our results thus highlight the importance of limiting crossover numbers to enable faithful chromosome segregation during sperm and egg development.

## Introduction

Meiosis is a specialized, reductional form of cell division necessary for the production of haploid sperm or egg cells. One hallmark of meiosis is the requirement of genetic exchange through recombination. Meiotic recombination is initiated by the formation of double strand DNA breaks (DSBs), which are repaired to form crossover (CO) and noncrossover events [1]. Germ cells in most organisms require a CO between homologous chromosomes to physically link each pair of homologs, enabling their proper segregation during the Meiosis I division. Despite the formation of many programmed DSBs, most organisms are limited in the number of COs formed, and formation of a CO tends to inhibit formation of other COs nearby on the same chromosome pair, a conserved phenomenon known as CO interference [2,3]. COs that are subject to interference are considered interfering COs (or Class I COs). Additionally, some organisms have a subset of COs that are not subject to interference (called “non-interfering”, or Class II COs), but these non-interfering COs represent only 5–35% of all meiotic COs in *A*. *thaliana*, *M*. *musculus*, and *S*. *cerevisiae* [1]. While CO interference is a well conserved phenomenon among most eukaryotes, the molecular consequences that result from the occurrence of multiple interfering COs are not clear. In addition, it is also unclear how these consequences may have contributed to the conservation of CO interference.

The model organism *Caenorhabditis elegans* is a particularly powerful system to study CO regulation because it exhibits remarkably strict CO control; under wild-type conditions, only one DSB per chromosome is repaired as a CO and all COs are interfering COs [4]. Several studies have demonstrated that even in the presence of an extreme excess of DSBs (10-fold greater than wild-type levels), only a single interfering CO, marked by the pro-crossover factor COSA-1, is made per pair of homologous chromosomes [5,6]. Additionally, it has been shown that CO interference can operate over distances longer than the length of a normal chromosome axis [5,7]; in the case of end-to-end fusions of chromosomes (that still accurately segregate in *C*. *elegans*), many meioses still only have one CO per fusion chromosome pair [5,7].

COs form physical connections between the homologs, known as a chiasmata. Since in *C*. *elegans* each chromosome pair typically has one CO that occurs off-center along the chromosome length, the chromosomes reorganize around this single chiasma to form cruciform bivalents with long and short arms [8,9]. These bivalents then align on the spindle, and in anaphase I, cohesion is lost along the short arm axis, enabling segregation of homologous chromosomes [10,11]. Aurora B kinase (AIR-2) and other members of the conserved chromosomal passenger complex (CPC) are targeted to this short arm region in prophase to protect sister chromatid cohesion in that region until anaphase I is triggered [8]. Moreover, the CPC also directs the formation of a larger meiotic protein complex that forms upon nuclear envelope breakdown [12,13]; at this stage, the CPC reorganizes from a linear distribution along the short arm axis to a ring encircling this region, and targets a number of other conserved proteins to form a structure known as the Ring Complex (RC) [14]. In addition to the CPC, the RC contains other conserved components, such as the kinase BUB-1 [13] and the microtubule de-stabilizing kinesin MCAK^KLP-7^ [15,16]. Furthermore, SUMO and SUMO pathway enzymes localize to the RC and are required for RC assembly and stability [17,18]. Chromosomes that lack RCs have congression and segregation errors [19], and depletion of RC components causes a variety of meiotic defects [12,13,15,18,20–23], highlighting the importance of this complex.

One function of the RC is to promote chromosome congression [12,18]. In *C*. *elegans* oocyte spindles, microtubule bundles run laterally along the sides of bivalents instead of forming canonical end-on kinetochore attachments. A component of the RC, the kinesin-4 family member KLP-19, has been proposed to walk along these laterally-associated bundles towards microtubule plus ends located in the center of the spindle, thus providing chromosomes with plus-end directed forces that mediate metaphase alignment [12]. Then, at anaphase onset, the enzyme separase (SEP-1) is targeted to the midbivalent region to cleave cohesin and allow homologous chromosomes to segregate to opposite spindle poles [19,24]. At this stage, the RCs are removed from chromosomes and remain in the center of the spindle, where they disassemble [13,17,19,25,26]. Oocytes extrude one set of homologs into a polar body, and then Meiosis II (MII) proceeds. The MII chromosomes assemble RCs encircling the sister chromatid interface and repeat the segregation process to form a matured haploid egg.

Although extensive research has focused on CO formation and RC function independently, it is still unclear how early meiotic processes affect chromosome structure and function in meiotic divisions. Moreover, why chromosomes in many organisms are so tightly restricted to 1–2 COs per homolog pair has not been addressed. Here we utilize *C*. *elegans* strains containing a chromosome that exhibits increased crossover numbers under specific conditions to assess the effects of supernumary crossovers on meiosis. We find that the RC is mispatterned in the presence of multiple COs, leading to defects in chromosome congression, and that chromosomes with multiple chiasmata exhibit extensive chromatin bridging in anaphase. Thus, excess crossovers can severely impact chromosome patterning and segregation, highlighting the importance of limiting the number of recombination events between homologous chromosomes for the proper execution of meiosis. Further, our studies uncovered multiple mechanisms by which oocytes are able to correct these errors, demonstrating that species have evolved ways to combat the deleterious defects caused by excess crossing over.

## Results

### Fusion chromosomes generate bivalents with multiple crossovers and chiasmata

**Fig 1:**
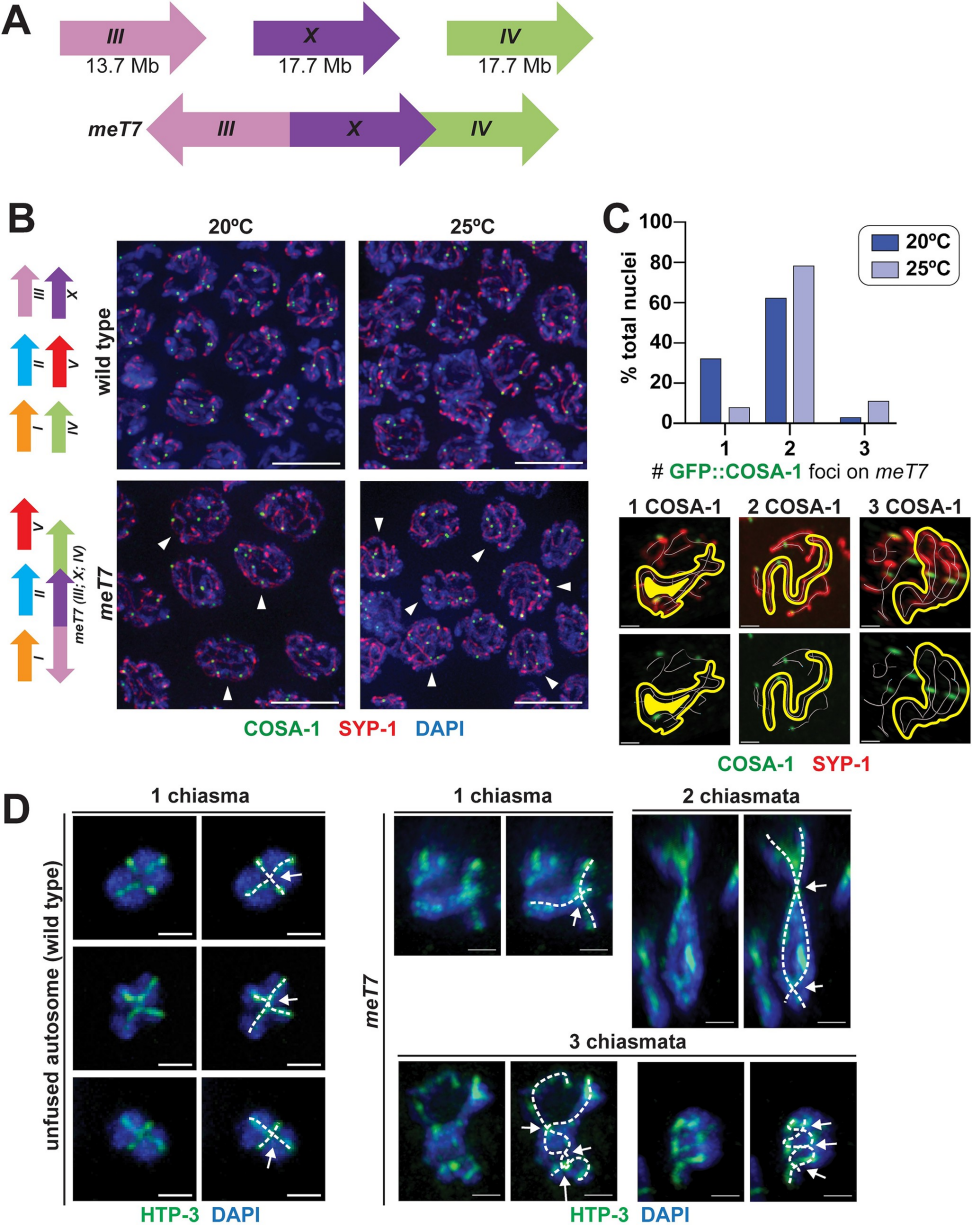
Fusion chromosomes generate bivalents with multiple crossovers and chiasmata. (A) Schematic indicating the orientation of the *meT7* fusion chromosome, which fuses the *X* chromosome and chromosomes *III* and *IV*. (B) Left, schematic depicting the chromosomes in a wild-type strain (top) and the *meT7 (X;III;IV)* fusion chromosome strain (bottom). Right, immunofluorescence images of GFP::COSA-1 in fixed late pachytene nuclei from wild-type and *meT7* fusion chromosome strains grown at 20°C and 25°C. GFP::COSA-1 is shown in green, synaptonemal complex protein SYP-1 is shown in red, and DNA is shown in blue. Arrowheads indicate nuclei with ≥2 crossovers along the *meT7* fusion chromosome. Scale bars = 5 μm. (C) Top, quantification of percentage of *meT7* nuclei with indicated number GFP::COSA-1 foci on *meT7* in late pachytene at 20°C and 25°C. Number of late pachytene nuclei scored for COSA-1 foci: 20°C, N = 290; 25°C N = 287. Bottom, representative immunofluorescence images of fixed single nuclei with *meT7* chromosomes with the indicated number of COSA-1 foci. White line indicates traced chromosome axis of each chromosome in a single nucleus. Yellow outline highlights the *meT7* fusion chromosome within the nucleus. (D) Three-dimensionally rendered immunofluorescence images of individual wild type and *meT7* diakinesis bivalents. Dashed lines (white) indicate traced HTP-3 axes (green), with crossing of axes and arrows indicating chiasmata. Scale bars = 1 μm.

To understand the effects of multiple COs on proper chromosome segregation and gamete formation, we sought to exploit the advantages of the *C*. *elegans* model system to consistently generate multiple COs along a single chromosome during meiosis. Since normal *C*. *elegans* chromosomes typically experience only one CO per meiosis [5,7], we utilized a strain containing the three-chromosome fusion *meT7* (end-on-end fusions of chromosomes *III*, *X*, and *IV*; Fig 1A) [7]. While wild-type strains contain six individual chromosomes, strains containing the *meT7* fusion chromosome have a total of four individual chromosomes total (*meT7 III; X; IV* fusion chromosome, and chromosomes *I*, *II*, and *V;* Fig 1A and 1B, and S1 Fig). When grown under standard conditions of 20°C, wild type and *meT7*-containing strains have relatively normal progeny viability (100% versus 98.8%; Table 1), indicating that *meT7* segregation errors leading to aneuploidy are rare [7]. Since *meT7* is three times the length of a normal chromosome, previous studies using genetic assays found the occasional occurrence of multiple COs along this fusion chromosome and that this chromosome is easily identifiable in meiotic nuclei due to its size [7,8].

**Table 1:** Progeny viability following *lem-3* RNAi treatment.

Genotype	RNAi Treatment	Total number of eggs laid (# broods evaluated)	% viable progeny	% inviable embryos
Wild type at 20°C	n/a	1,003 (4)	100	0
Wild type at 25°C	n/a	2,483 (15)	99.5	0.5
*GFP*::*TBB-2 mCh*::*HIS-11* at 25°C	n/a	2,303 (15)	98.4	1.6
*meT7 (III;X;IV)* at 20°C	n/a	869 (9)	98.8	1.2
*meT7 (III;X;IV)* at 25°C	n/a	1,196 (15)	72.4	27.6
*meT7 (III;X;IV) GFP*::*TBB-2 mCh*::*HIS-11* at 25°C	n/a	1,230 (15)	63.2	36.8
Wild type at 25°C	*lem-3*	625 (5)	98.6	1.4
*GFP*::*TBB-2 mCh*::*HIS-11* at 25°C	*lem-3*	533 (5)	98.1	1.9
*meT7 (III;X;IV)* at 25°C	*lem-3*	1,256 (10)	66.1	33.9
*meT7 (III;X;IV) GFP*::*TBB-2 mCh*::*HIS-11* at 25°C	*lem-3*	582 (10)	45.4	54.6

To determine the exact number of Class I interfering COs occurring along the length of *meT7* within a population of nuclei, we assessed the number of COSA-1 foci along *meT7* chromosomes in single nuclei of strains grown in standard conditions; COSA-1 is a conserved protein required for CO formation that acts with other pro-crossover factors (MSH-5, ZHP-1, ZHP-2, ZHP-3, and ZHP-4) and serves as a robust cytological marker of Class I interfering COs during late pachytene [6,27–29]. In wild-type strains containing unfused chromosomes, the six individual chromosomes in each nucleus obtain a single CO per chromosome resulting in 6 COSA-1 foci per nucleus, with <0.4% of nuclei obtaining more than 6 foci [5,6] (Fig 1B and Materials and Methods). In contrast, strains grown at 20°C with the *meT7* fusion chromosome obtain 4–6 COSA-1 foci per late pachytene nucleus (Fig 1B arrowheads), with 77% of *meT7* chromosomes exhibiting ≥2 COSA-1 foci, consistent with previous analysis of *meT7* chiasmata [8] (Fig 1C). To further increase the number of COs occurring along *meT7*, we grew strains containing the *meT7* fusion chromosome at 25°C, a temperature that was previously found to increase the number of COs along the length of the two-chromosome fusion *mnT12* [5] (S1 Fig and S2 Fig). Compared to 20°C, we found that *meT7* strains grown at 25°C had an increase in COSA-1 foci, with >90% of *meT7* chromosomes with 2 or more COSA-1 foci (Fig 1B arrowheads and 1C; 77% versus 90%; p<0.0001; Mann Whitney test, two-tailed) and an increase in progeny inviability (1.2% versus 27.6%; p<0.0001; Fisher’s exact test, two-tailed; Table 1). This enrichment of COSA-1 foci in *meT7* strains indicates the occurrence of multiple COs along the length of the *meT7* fusion chromosome.

To determine whether these increased COSA-1 foci along *meT7* fusion chromosomes represent COs that become chiasmata, we assessed bivalent structure and chiasma number at diakinesis along fusion chromosome pairs (Fig 1D and S3 Fig). COs are normally formed off-center of the *C*. *elegans* chromosome and some meiotic chromosome proteins reorganize around these CO sites starting at the late pachytene-diplotene transition, resulting in cytologically distinguishable long and short bivalent arms; in this structure, the chiasma is positioned where the chromosomes axes cross [8,9,30] (Fig 1D; chromosome axes marked by HTP-3 immunofluorescence). In accordance with previous analysis performed at 20°C [8], we found that some *meT7* bivalents grown at 25°C exhibit unique meiotic chromosome structural reorganization, which is consistent with the occurrence of multiple COs. Notably, some *meT7* bivalents exhibited multiple chiasmata (Fig 1D, chiasmata indicated with arrows) and multiple short arms (S3 Fig, long and short arms marked with HTP-1/2 and SYP-1, respectively); we also observed similar structures in the two-chromosome fusion *mnT12* [8] (S3 Fig). While unfused chromosomes at diakinesis only had one chiasma (21/21 at 20°C and 21/21 at 25°C), we found that 13/21 diakinesis nuclei with *meT7* bivalents at 20°C had multiple chiasmata, consistent with 60–80% of the *meT7* chromosome pair having more than one COSA-1 focus at the late pachytene stage (Fig 1C). In *meT7* strains grown at 25°C, we found that 19/21 (90.5%) of *meT7* fusion chromosomes had more than one chiasma, consistent with 90% of *meT7* chromosome pairs having 2 or more COSA-1 marked crossovers (Fig 1C) as well as a temperature-dependent increase in crossovers that result in chiasmata (20°C versus 25°C; P = 0.02; Mann-Whitney, two-tailed). Together, this corresponding increase in both COSA-1 foci and chiasmata indicates that the temperature-associated extra COSA-1 foci observed at pachytene in *meT7* chromosomes represent *bona fide* cytologically-differentiated meiotic CO events that can result in atypical bivalent structures.

### Bivalents with multiple crossovers have mispatterned Ring Complexes

Given that we can increase CO numbers along an easily identifiable chromosome under a specific condition, we set out to investigate how extra chiasmata might affect other aspects of bivalent organization by assessing the Ring Complex (RC), a structure comprised of a set of critical meiotic proteins. AIR-2 and other CPC components localize along the short arms of each cruciform bivalent during diakinesis, and upon nuclear envelope breakdown, the CPC reorganizes into a ring encircling the short arm axis of the bivalent, and forms the RC by recruiting other components [12,13,18]. To determine if this structure properly forms on bivalents with multiple short arm regions (resulting from excess COs) (Fig 1D), we compared the organization of the CPC and other RC components on wild-type and *meT7* prometaphase bivalents.

**Fig 2:**
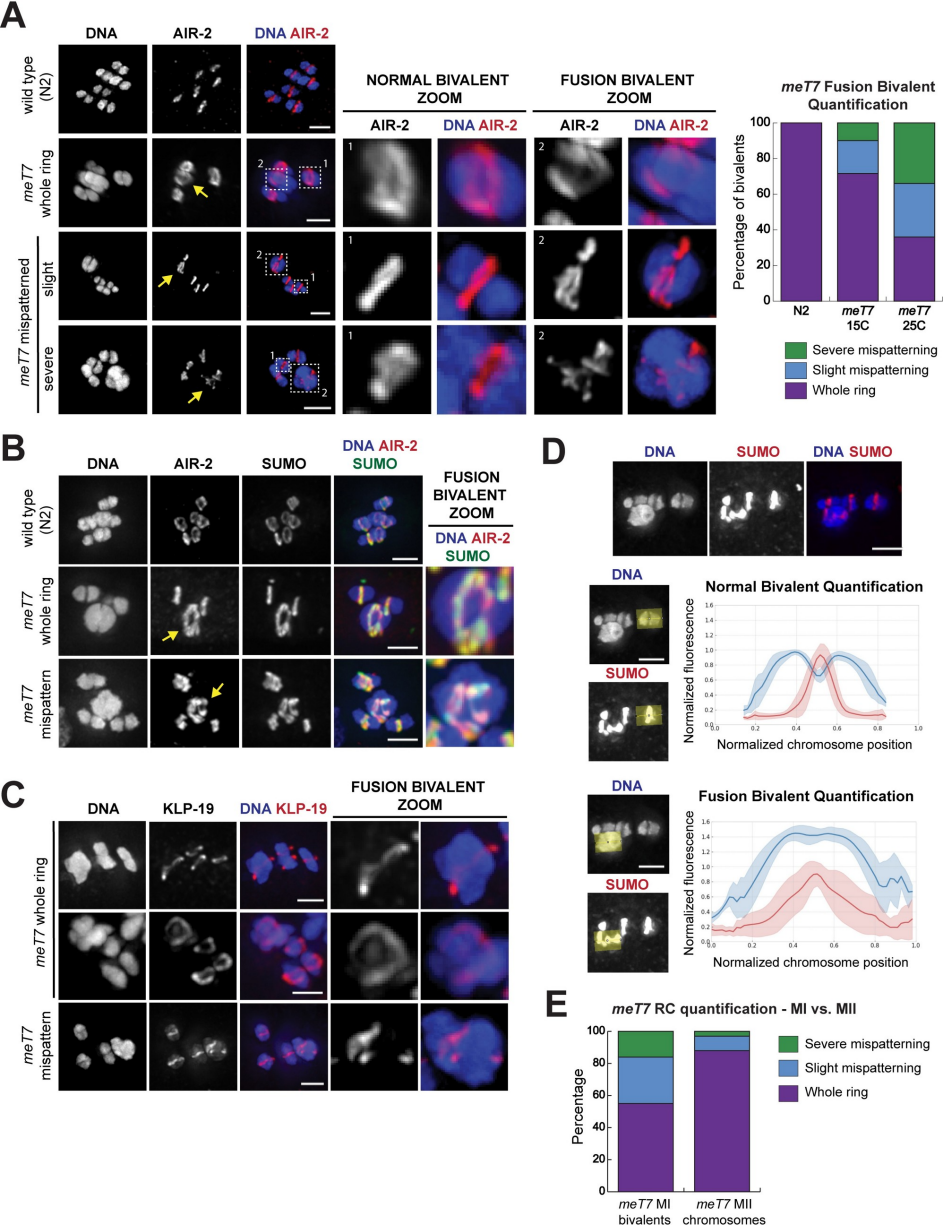
Bivalents with excess crossovers have mispatterned Ring Complexes (RCs). (A) Immunofluorescence of AIR-2 localization in fixed wild type (N2) or *meT7* oocytes. Single-chromosome zooms show that AIR-2 (red) localizes in a whole ring shape encircling normal bivalents in both strains, while its localization is either whole ring-like or mispatterned on *meT7* fusion bivalents (yellow arrows). Note that the zoomed images throughout this figure are partial projections, chosen to highlight individual chromosomes. Quantification in graph to the right of the images (N = Whole ring/Slight/Severe: N2 at 15°C N = 31/0/0, N2 at 25°C N = 44/0/0, *meT7* at 15°C N = 54/14/7, *meT7* at 25°C N = 27/23/25). This analysis shows that *meT7* mispatterning increases with increased temperatures. For overall mispatterning (slight + severe): 15°C (21/75) versus 25°C (48/75), P = 0.0001; Fisher’s exact, two-tailed. For severe mispatterning: 15°C (7/75) versus 25°C (25/75), P = 0.0006; Fisher’s exact, two-tailed. (B) SUMO (green) co-localizes with AIR-2 (red) in all ring structure types on both fixed wild type (N2) and *meT7* fusion bivalents; RCs on *meT7* bivalents denoted with yellow arrows. (C) KLP-19 (kinesin 4) (red), localizes to both “whole ring” and “mispatterned” *meT7* RCs in fixed oocytes. (D) Linescans across bivalents of fixed oocytes show that both the bivalent and RC have wider spread and higher variance in *meT7* fusion bivalents as compared to wild-type bivalents. (N = 25, all categories). (E) Quantification of ring mispatterning in metaphase I bivalents (34/75, 45.3%) and metaphase II sister chromatid pairs (9/75, 12.0%). Metaphase I bivalents have much higher rates of mispatterning as compared to metaphase II sister chromatid pairs (P<0.0001, Fisher’s exact test, two-tailed). All scale bars = 2.5μm.

The three wild-type bivalents in the *meT7* strain formed single whole rings of AIR-2 (75/75 had whole rings at 15°C; 74/75 at 25°C). However, while AIR-2 localized to *meT7* fusion bivalents, it was sometimes improperly shaped: 72.0% of *meT7* bivalents had single rings at 15°C, but other *meT7* bivalents formed either slightly (18.7%) or severely (9.3%) mispatterned structures (Fig 2A and 2B; S4 Fig; “slight mispatterning” denotes that ring components are localized to a single plane but are not confined to a single ring around the outside of the bivalent, while “severe mispatterning” indicates that components form more complex structures). Additionally, we also observed similar types of ring mispatterning along the two chromosome fusion *mnT12* (S5A Fig). BIR-1^Survivin^, another CPC component, colocalized with AIR-2 on all ring structure types (S6 Fig), suggesting that the pattern of AIR-2 localization reflects assembly of the entire CPC. Similar to the increase in COs and chiasmata at 25°C along *meT7* (77% *meT7* with >2 COs at 20°C versus 90% *meT7* with >2 COs at 25°C; Fig 1D), these CPC patterning defects on the *meT7* bivalent increased in number (21/75 total mispatterned rings at 15°C versus 48/75 total mispatterned rings at 25°C, P = 0.0001; Fisher’s exact, two-tailed) and severity (observed severely mispatterned ring frequency was 9.3% at 15°C versus 33.3% at 25°C, P = 0.0006; Fisher’s exact, two-tailed) with increased temperatures (Fig 2A), suggesting that increased CO numbers increase the likelihood of CPC mispatterning.

We next assessed the localization of RC components that are dependent on the CPC for targeting, and found that both SUMO (Fig 2B) and KLP-19 (Fig 2C) localize on *meT7* bivalents. Therefore, although the CPC is mispatterned, other RC components can still target to the bivalents. Linescans across SUMO-stained bivalents showed that, in contrast to the single tight ring peak and bilobed bivalent structure characteristic of wild-type bivalents, *meT7* bivalents have broad variation in both RC and chromosome shape (Fig 2D). Importantly, although RCs also form in Meiosis II around the interface between sister-chromatids, the RC patterning defects were more prevalent on Meiosis I *meT7* bivalents (45.3% of all MI *meT7* bivalents, compared to 12.0% of MII chromosomes, p<0.0001, Fisher’s exact test, two-tailed; Fig 2E). This result is consistent with the idea that the RC defects are primarily caused by excess COs between homologous chromosomes, rather than general problems with RC formation in the *meT7* strain.

Next we assessed kinetochore organization on *meT7* bivalents. In *C*. *elegans*, kinetochore proteins coat the holocentric chromosomes in meiosis, forming a cup-like structure around each end of the bivalent [31,32]. Although microtubules run along the sides of chromosomes and do not form end-on kinetochore attachments [12], kinetochores help orient bivalents within the spindle [13], such that the long axis of the bivalent is parallel to the long axis of the spindle; this arrangement ensures that the two homologs are pointed towards opposite spindle poles, with the short-arm axis, where cohesion will be released, in the middle. We found that two kinetochore components, BUB-1 and SEP-1^Separase^, still localized to the *meT7* fusion chromosome bivalent (S7A Fig and S7B Fig), suggesting that the targeting of kinetochore proteins to *meT7* was not affected. However, the fusion chromosome bivalents are often misshapen and many do not have a clear long axis (S7C Fig and example images throughout the paper). This altered bivalent shape would therefore prevent *meT7* from being able to properly assemble the two distinct cup-like kinetochores, causing orientation problems for the *meT7* bivalent within the spindle.

### Fusion chromosome bivalents can form multiple Ring Complexes that act independently of one another

**Fig 3:**
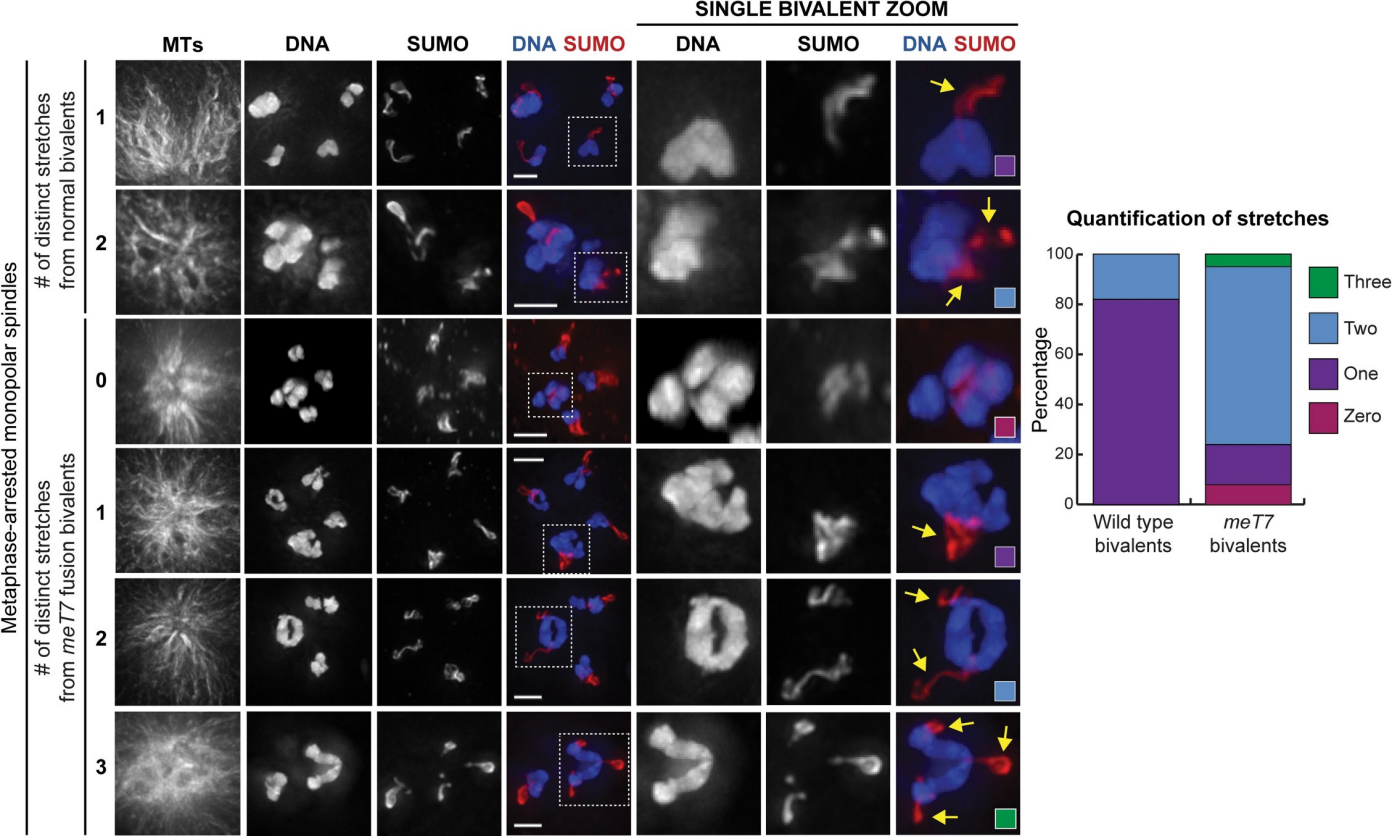
Bivalents with excess COs can form multiple RCs. Examples and quantification of immunofluorescence images of stretching RCs (visualized by SUMO staining, red) from bivalents (blue) of fixed oocytes (N = 38, all conditions). In this assay, monopolar spindles were generated by depleting the force-generating motor KLP-18 [12,68] and metaphase I arrest was achieved by depleting anaphase promoting complex component EMB-30. Zooms are partial projections, chosen to highlight individual chromosomes. RCs on monopolar spindles under prolonged metaphase arrest tend to stretch in a plus end-directed manner, towards the outside of the microtubule aster. RCs on normal bivalents tend to stretch as mostly one unit in a single direction, while *meT7* bivalents can have multiple RCs that stretch in independent directions. Yellow arrows denote stretching RCs. Scale bars = 2.5μm.

Next, we wanted to characterize the mispatterned *meT7* RC structures. In some of our images there appeared to be multiple distinct RCs forming on a single *meT7* bivalent (e.g. Fig 2A, row 3; Fig 2B, row 3), which could reflect the ability of each chiasma to organize its own RC. However, since many of the mispatterned RCs appeared to be complex structures, it was difficult in many cases to determine if a particular RC was comprised of multiple rings close together, or instead represented a single intertwined structure. Therefore, to distinguish between these possibilities, we performed an “RC stretching assay.” This assay exploits our previous finding that under extended metaphase arrest, RCs begin to stretch away from the chromosomes towards microtubule plus ends, which we interpret to mean that they contain a plus-end-directed activity that in normal conditions would provide chromosomes with plus-end-directed forces [19]. For the purposes of the current study, we thought that this behavior might spatially separate distinct rings from one another, enabling us to distinguish and quantify the total number of rings formed on each *meT7* fusion bivalent. Additionally, we performed this “RC stretching assay” on monopolar spindles, in which the microtubule minus ends are organized at a central pole and the plus ends radiate outward forming an aster. We reasoned that this feature might enable us to determine if the *meT7* ring structures were functional (*i*.*e*. whether they were able to stretch towards the outside of the aster, thus exerting plus-end forces). Consistent with previous work [19], we found that in this RC stretching assay, rings on normal bivalents typically stretch off as one entity in a single direction towards microtubule plus ends (31/38), with 7/38 of the observed bivalents having two stretches (Fig 3, rows 1 and 2). In contrast, on many *meT7* bivalents, it was clear that there was more than one RC, often with two (27/38) or rarely three (2/38) separate entities stretching off of the bivalent (Fig 3, rows 5 and 6). Notably, the frequency of more than one stretching RC on the *meT7* fusion chromosome (76% at 15°C) is close to the frequency of more than one CO along *meT7* (77% at 20°C; Fig 1C). This result suggests that multiple distinct RC structures can form at different chiasmata on the same bivalent.

Interestingly, when multiple RCs are present, they stretch towards microtubule plus ends but end up oriented along different microtubule bundles, suggesting that these RCs are acting independently of one another; in the context of a bipolar spindle where microtubule bundles are not all in the same orientation, this could result in a single chromosome being pulled in opposite directions. Furthermore, unlike on normal bivalents, 3/38 rings on *meT7* bivalents were not stretching in any direction (the “zero stretches” category in Fig 3, row 3), despite normal bivalents in the same spindle having stretching rings, suggesting that mispatterning may sometimes affect RC function. Together, these results suggest that the mispatterned RCs on *meT7* may not be able to provide chromosomes with normal plus-end-directed forces.

### Fusion chromosome bivalents show defects in metaphase alignment

**Fig 4:**
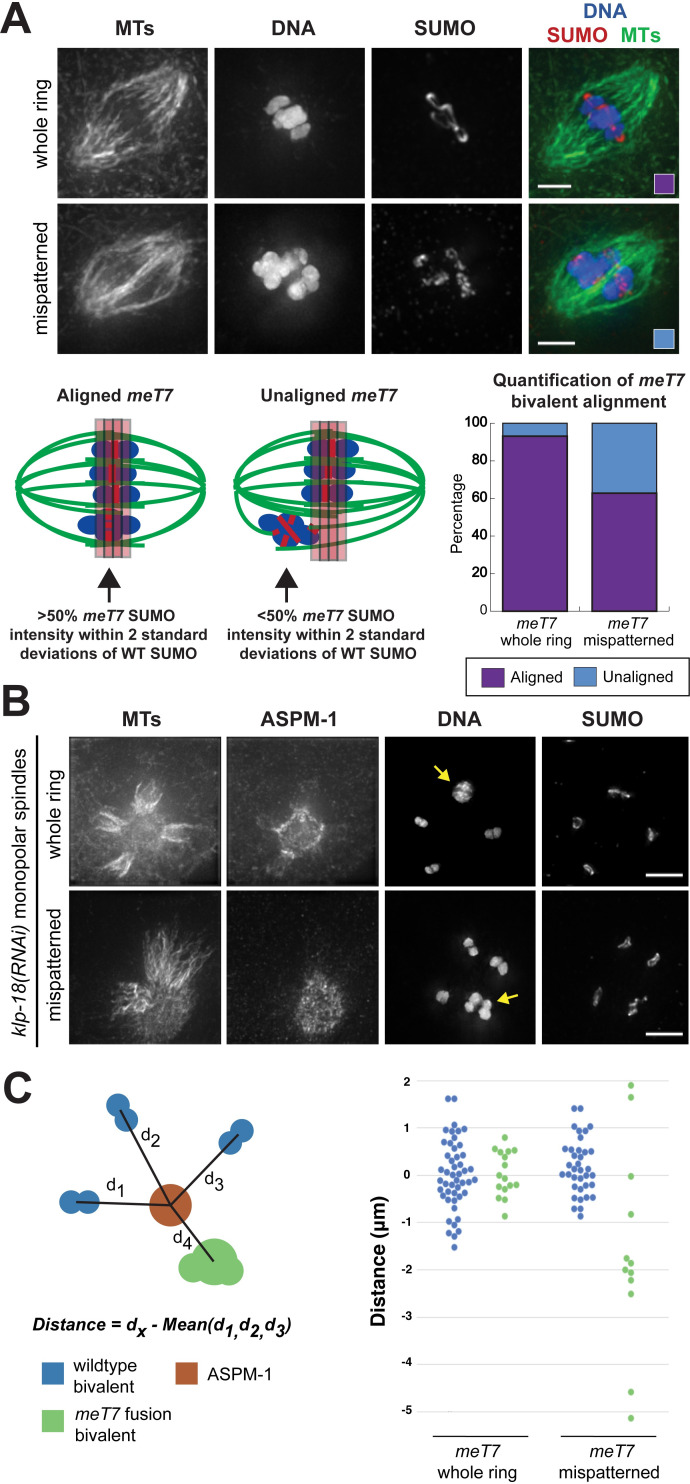
Bivalents with mispatterned RCs align improperly on the oocyte spindle and are not subjected to proper plus-end directed forces. (A) Immunofluorescence images of fixed oocytes show DNA (blue), microtubules (green) and SUMO to mark the RCs (red). Alignment of the fusion chromosome was assessed on spindles where the three normal bivalents were aligned; note that alignment was assessed by rotating the images in 3D, since this is harder to discern in projection images, like those shown. A diagram showing our quantification scheme and our quantification is below the images. 92% of *meT7* bivalents with a whole ring aligned with the three normal bivalents, compared to 62% of *meT7* bivalents with mispatterned rings (N = 50, all conditions). Scale bars = 2.5μm. (B,C) Examples and quantification of fused chromosome bivalent position in fixed oocytes; *meT7* bivalents indicated by arrows. Monopolar spindles were generated by depleting KLP-18, and the center of the aster was determined by staining with spindle pole marker ASPM-1 and determining the center of the ASPM-1 signal. The distance between each of the bivalents and the center of the ASPM-1 signal was measured using Imaris, and the distance of the fused chromosome bivalent was compared to the average of the three normal bivalents’ distance; normal bivalents were also assessed with this same measure (comparing the position of each individual bivalent with the average of the three normal bivalents on the same spindle). On the graph, blue dots denote wild-type bivalents, and green dots denote *meT7* bivalents; these measurements were derived from monopolar spindles where the *meT7* bivalent was present in a whole ring (left side of graph) or where the RC was mispatterned (right side of graph). *meT7* bivalents with whole rings (N = 16) tended to be positioned similarly to normal bivalents on the monopole (P = 0.90; Mann-Whitney, two-tailed), while bivalents with mispatterned rings (N = 12) showed much more variation in their distance from the monopole as compared to the normal bivalents (P = 0.002; Mann-Whitney, two tailed). Scale bars = 5μm.

To investigate the possibility that *meT7* bivalents are experiencing abnormal forces, we asked whether mispatterning of the RCs in the *meT7* bivalents had functional consequences on the alignment of those bivalents on bipolar spindles. We therefore evaluated spindles where the three normal bivalents had aligned at the metaphase plate and scored the position of the *meT7* fusion bivalent. We found that while *meT7* bivalents with single whole rings aligned with the other bivalents in 92% (46/50) of these spindles, only 62% (31/50) with mispatterned rings successfully aligned (Fig 4A); this correlation suggests that the alignment defects are not caused by the large size of *meT7* but by defects in RC organization, including the acquisition of multiple RCs on the *meT7* bivalent (Fig 3). Since this is fixed imaging, we do not know if this phenotype represents a complete failure of *meT7* to align, or just a delay compared to the other bivalents. However, either way our data suggest that the acquisition of multiple crossovers on the *meT7* bivalent affects the efficiency of chromosome congression.

Building on this finding, we assessed the position of chromosomes on monopolar spindles as a read-out of plus-end directed forces (Fig 4B); in this context, bivalents that are able to generate normal plus-end forces migrate away from the center of the aster [12]. To define this center, we used ASPM-1 as a pole marker, and measured the distance from the center of each bivalent to the center of the ASPM-1 staining (Fig 4B and 4C). Unlike the RC stretching assay with monopolar spindles (Fig 3), this experiment does not involve an extended metaphase arrest so the RCs retain their original morphology (Fig 4). This analysis revealed that *meT7* bivalents with mispatterned rings do not tend to migrate as far away from the pole as the three wild-type bivalents (P = 0.002; Mann-Whitney *U* test, two tailed, Fig 4C). Moreover, in two extreme cases (out of 12), the fusion bivalents remained stuck at the center of the monopole (Fig 4B, bottom row). Importantly, this defect appeared to be caused by the chromosome patterning defects and not by the large size of the fusion bivalent, since *meT7* bivalents with single rings exhibited normal chromosome positioning (Fig 4B and 4C). Together, these findings suggest that the mispatterned RCs on *meT7* bivalents are not able to provide these bivalents with normal plus-end forces, potentially impacting their ability to achieve proper metaphase alignment. Since *meT7* bivalents often have multiple RCs that can function independently of each other (Fig 3), we postulate that these RCs could provide bivalents with forces in opposing directions thereby reducing the efficiency of chromosome movement.

### Fusion chromosome bivalents show defects in chromosome segregation that carry over into Meiosis II

**Fig 5:**
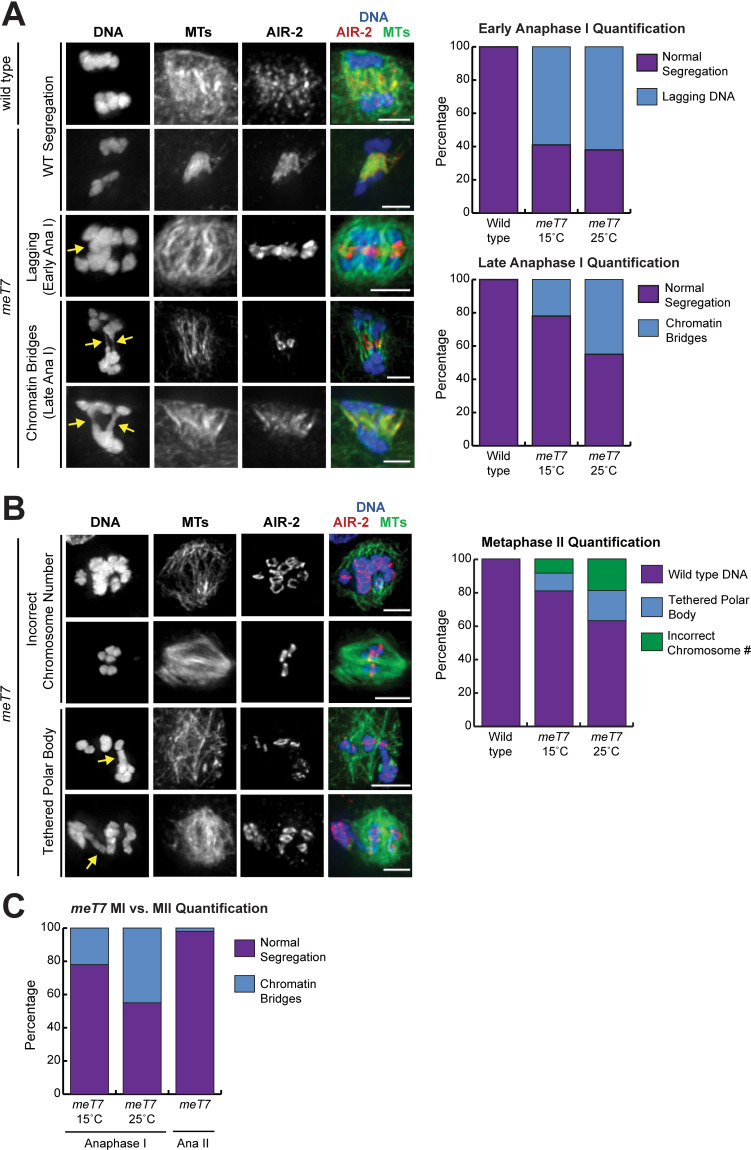
Fusion chromosome segregation is frequently aberrant. (A) Immunofluorescence of anaphase I chromosome segregation in fixed wild-type (N2) and *meT7* oocytes; shown are DNA (blue), microtubules (green), and AIR-2 (red), with quantification to the right of the images. All chromosomes (N = 75 for early and mid-to-late anaphase) segregated without errors in wild type (N2) oocytes (rows 1 and 2), but *meT7* bivalents were often found in the center of the spindle in early anaphase when the normal bivalents were segregating (row 3, indicated with arrow, “early anaphase quantification” graph), suggesting a segregation delay. This phenotype was not different in *meT7* oocytes between 15°C and 25°C (P = 0.738, Fisher’s exact test, two-tailed). In mid-to-late anaphase, 17/75 (22.7%) of *meT7* bivalents showed extended chromatin bridging, indicated by arrows, at 15°C, which increased to 34/75 (45.3%) at 25°C (rows 4 and 5, “late anaphase quantification” graph; P = 0.0056, Fisher’s exact test, two-tailed). (B) Metaphase II spindles in *meT7* oocytes. While all N2 metaphase II spindles were euploid (N = 90), at 15°C 18/90 (20.0%) of *meT7* spindles were either aneuploid (rows 1 and 2) or had DNA tethered to the first polar body (indicated with arrows, rows 3 and 4). At 25°C this frequency was 33/90 (36.6%), suggesting that these defects increase at higher temperature (P = 0.02, Fisher’s exact test, two-tailed). (C) Quantification of *meT7* chromatin bridging in anaphase I vs. anaphase II. 22.5% (16/71) of mid-to-late *meT7* anaphase I spindles at 15°C and 44.8% (26/58) at 25°C showed anaphase chromatin bridging, while only 3.1% of all *meT7* anaphase II spindles (2/65; both temperatures combined) showed anaphase chromatin bridging. All scale bars = 2.5μm.

Given the defects in the structure and alignment of *meT7* bivalents, we next wanted to determine if having excess COs impacts chromosome segregation during Meiosis I and II. First, we imaged anaphase I to assess the ability of homologous chromosomes to segregate. Notably, we found that in early anaphase I, the *meT7* fusion bivalent was often present in the middle of the spindle in cases where the three wild-type bivalents were segregating, suggesting that its segregation was delayed relative to the other chromosomes. However, the frequency of this phenotype did not appear to be affected by temperature, as *meT7* bivalents showed comparable segregation delays in early anaphase I spindles at both 15°C (44/75, 58.7%) and 25°C (47/75, 62.7%) (P = 0.738, Fisher’s exact test, two-tailed, Fig 5A). In contrast, in late anaphase I, *meT7* bivalents had chromatin bridges that increased at higher temperatures where *meT7* experiences elevated COs (17/75, 22.7% at 15°C vs. 34/75, 45.3% at 25°C; P = 0.0056, Fisher’s exact test, two-tailed), while wild-type bivalents segregated normally at all temperatures (Fig 5A). Similarly, the *mnT12* two chromosome fusion bivalents also experience late anaphase I chromatin bridges (S5B Fig). Collectively, these results suggest that the early anaphase I defects are not due to increases in CO numbers, and may instead be due to the large size of the fusion chromosome. Conversely, extra COs between homologous chromosomes likely cause chromatin bridges in late anaphase I. Consistent with this hypothesis, we rarely observed chromatin bridging in anaphase II when sister chromatids rather than homologous chromosomes were segregating (2/65, 3.1%; Fig 5C).

To determine if the anaphase I defects had lasting consequences, we examined oocytes that progressed to Meiosis II. Notably, we observed a range of severe defects at 15°C, from DNA connecting the polar body to chromatin in the MII spindle (“tethered polar body” category; Fig 5B, arrows) to either gain or loss of the entire *meT7* bivalent. Similar to the chromosome bridging observed in MI, these defects increased at the higher temperature of 25°C (20%, 18/90 at 15°C versus 36.6%, 33/90 at 25°C; P = 0.02, Fisher’s exact test, two-tailed; Fig 5B), suggesting that these defects were the result of excess COs between *meT7* chromosomes. We also observed Meiosis II defects in the *mnT12* strain (S5C Fig), demonstrating that these observations are not specific to *meT7*. The frequency of the severe defects seen for *meT7* at Meiosis II (33/90, 36.6% at 25°C) is close to the frequency of embryonic lethality seen in the *meT7* strain at 25°C (27.6%; Table 1), suggesting the majority of the embryonic lethality is due to a combination of aneuploidy and/or polar body extrusion defects.

### Persistent chromatin bridges can be recognized and resolved by the oocyte

**Fig 6:**
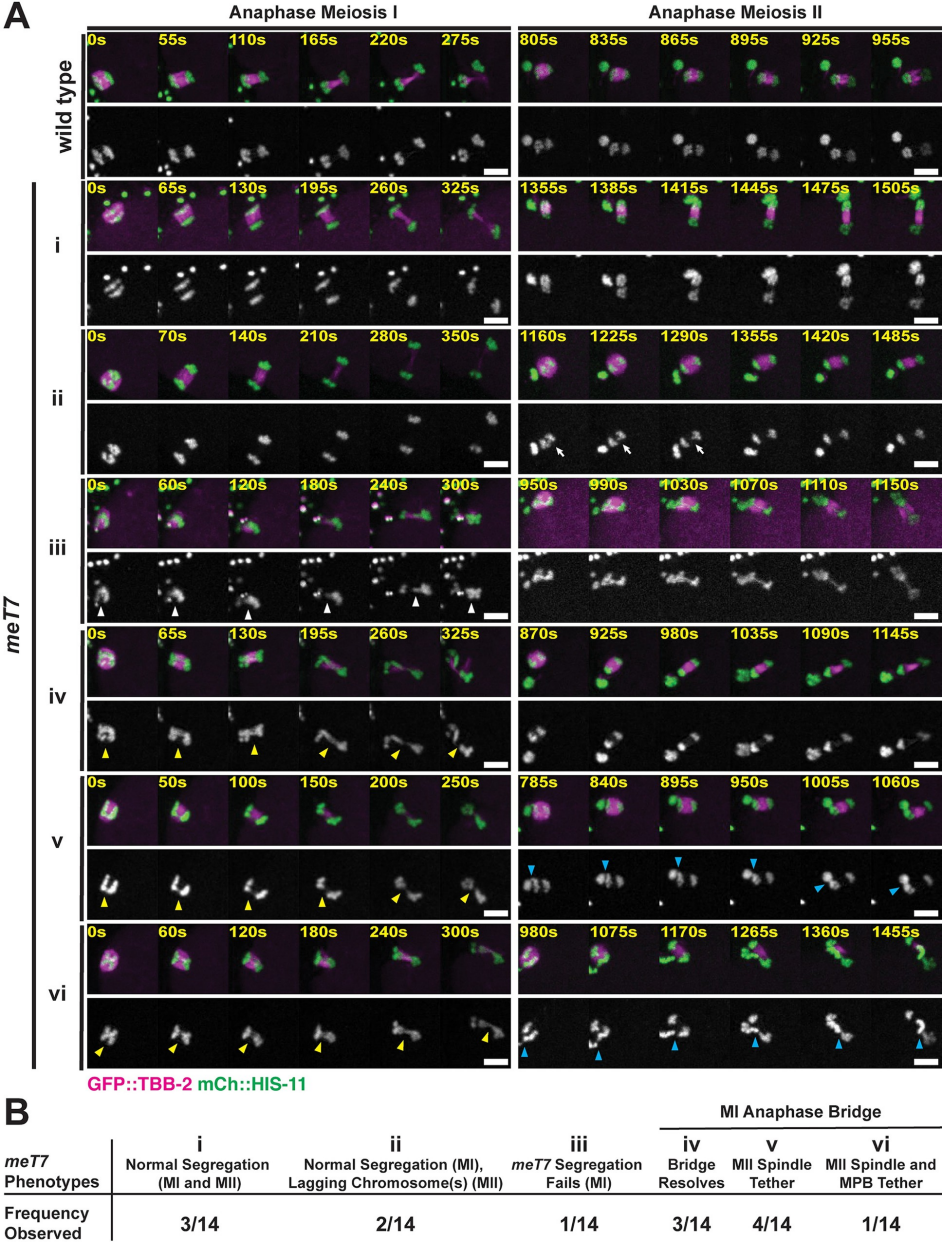
*meT7* oocytes display several classes of chromosome segregation defects. (A) Time-lapse spinning disk confocal montages during anaphase Meiosis I (MI) and anaphase Meiosis II (MII) in live wild-type and *meT7* oocytes expressing GFP::TBB-2 and mCherry:HIS-11 to mark microtubules and histones, respectively. Examples of *meT7* chromosome segregation phenotypes shown in i-vi. White arrowheads point to *meT7* fused chromosomes as they fail to segregate in anaphase I (iii), yellow arrowheads indicate anaphase I *meT7* chromatin bridges between segregating chromosomes (iv, v, vi), and blue arrowheads point to chromatin tethers between the MI polar body (MPB) and the anaphase II spindle (v, vi). White arrows indicate lagging chromosome(s) in anaphase II (ii). Time zero is the time at which chromosome segregation appears to begin. Scale bars = 5μm. (B) *meT7* chromosome segregation phenotype descriptions and observation frequency. Of the 8 *meT7* oocytes that had chromatin bridges at anaphase I, 3/8 bridges were able to resolve prior to the end of anaphase I. For the remaining 5 *meT7* oocytes that were not able to resolve their anaphase I bridges, 4/8 extruded the tether (with the associated chromatid) into the polar body at anaphase II and 1/8 exhibited persistent tethers through anaphase II.

Despite the fact that we observed meiotic chromosome segregation defects (Fig 5), the embryonic lethality of the *meT7* strain is surprisingly low (Table 1). The *meT7* strain exhibits 27.6% embryonic lethality at 25°C (Table 1), indicating that most oocytes are able to generate viable progeny even in the presence of multiple COs (at 25°C, >90% of *meT7* chromosomes have ≥2 COSA-1 foci; Fig 1C) and chromosome segregation defects (at 25°C, ~50% of *meT7* anaphase I nuclei have chromatin bridges; Fig 5). Similarly, Meiosis II oocytes have a lower frequency of errors than was observed during Meiosis I. Therefore, it is possible that the anaphase I bridges could in some cases be resolved (*i*.*e*. not persist into Meiosis II) in a manner that enables viability. To investigate this possibility, we performed live imaging of the meiotic divisions using a *meT7* strain expressing GFP-tubulin and mCherry-histone to mark microtubules and chromosomes, respectively. Confirming our fixed imaging results, we found that a majority of anaphase I spindles displayed extended chromatin bridges (8/14, 57%) (Fig 6). In some cases (5/8) these bridges persisted and either 1) the first polar body was tethered to the developing Meiosis II spindle (S1 Movie), or 2) *meT7* was not able to segregate in anaphase I, and the fully retained bivalent segregated with a chromatin bridge in anaphase II (S2 Movie). This supports the conclusion that oocytes face severe chromosome segregation defects in the presence of supernumerary crossovers. However, our live imaging also revealed instances where anaphase I DNA bridges were resolved as the oocyte progressed to Meiosis II (3/8; Fig 6; S3 Movie). Thus, oocytes appear to have mechanisms to correct and resolve chromosome segregation defects.

**Fig 7:**
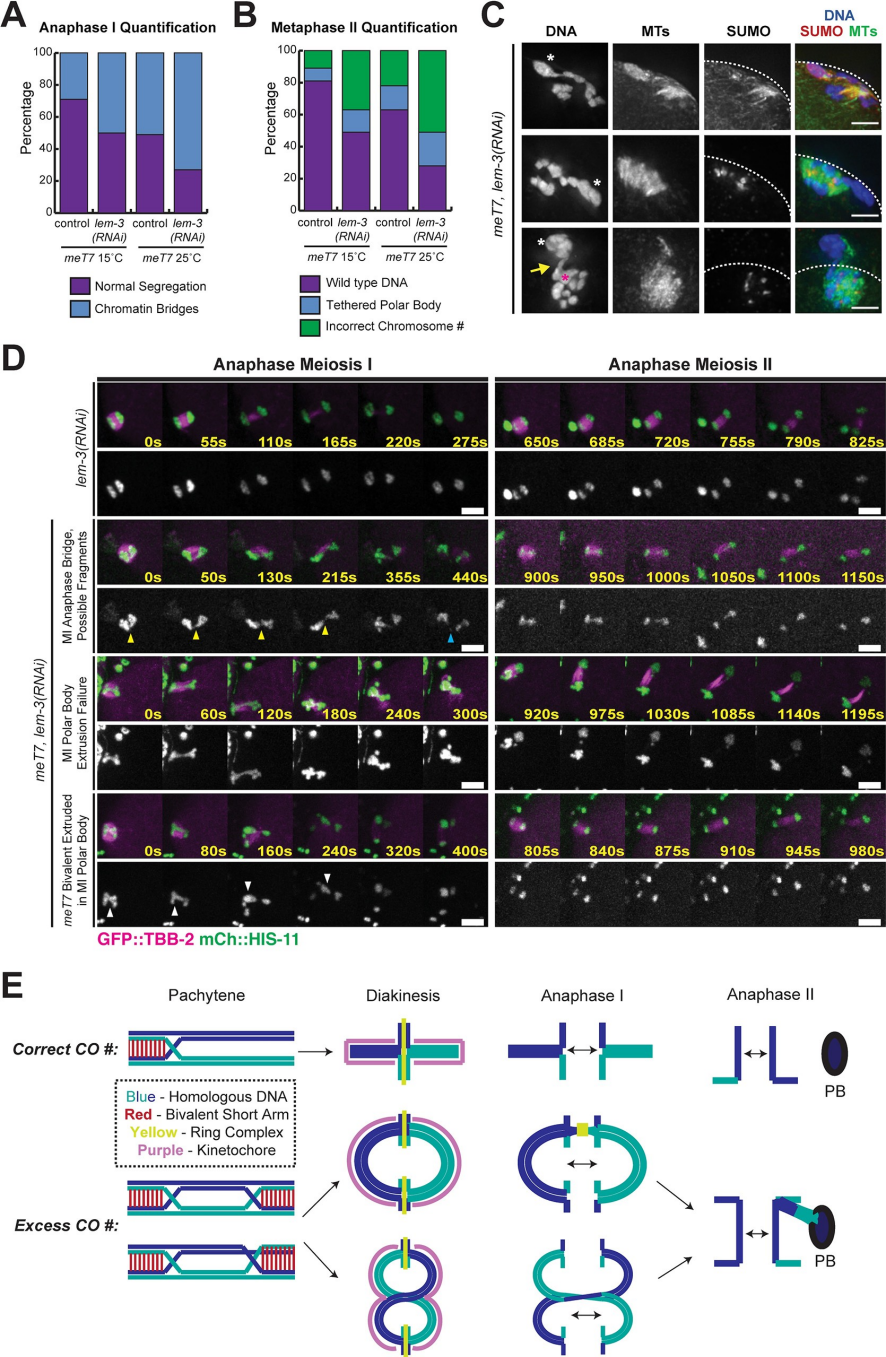
Oocytes can correct errors from excess COs in anaphase. (A) Frequency of anaphase bridges in vector control (N = 52 at 15°C; N = 49 at 25°C) and *lem-3(RNAi)*-treated (N = 52 at 15°C; N = 37 at 25°C) *meT7* anaphase I oocytes. Frequency of anaphase I bridges on *meT7* increased following *lem-3(RNAi)* at 25°C (51.0%, 25/49 for control compared to 72.97%, 27/37 for *lem-3(RNAi)*; P = 0.047, Fisher’s exact test, two-tailed). (B) Frequency of oocytes with DNA connecting the polar body and the MII spindle (“tethered polar body” category) or anueploidy in vector control (N = 48 at 15°C; N = 41 at 25°C) and *lem-3(RNAi)*-treated (N = 31 at 15°C; N = 28 at 25°C) metaphase II *meT7* oocytes. Frequency of polar body extrusion delays or aneuploidy increased at 25°C (36.6%, 15/41 for control compared to 71.4%, 20/28 for *lem-3(RNAi)*; P = 0.007, Fisher’s exact test, two-tailed). (C) Immunofluorescence of fixed anaphase II *meT7* oocytes treated with *lem-3(RNAi);* shown are DNA (blue), microtubules (green), and SUMO (red). In 23/26 of *lem-3(RNAi)* oocytes in which the polar body tether (yellow arrow, bottom row) persisted into anaphase II, the tethered sister chromatid (*meT7*, magenta asterisk in bottom row) appeared to be segregating to the cell cortex (three examples shown, polar bodies denoted with white asterisks). Scale bars = 2.5μm. (D) Time-lapse montages of anaphase I and II in live *lem-3* depleted wild type and *meT7* oocytes expressing GFP::TBB-2 (microtubules) and mCherry::HIS-11 (histones). Yellow arrowheads indicate an anaphase I chromatin bridge. Blue arrowhead denotes a possible chromosome fragment after bridge breaking. White arrowheads point to a failure to segregate *meT7* in Meiosis I and extrusion into the first polar body. Time zero is the time at which chromosome segregation appears to begin. Scale bars = 5μm. (E) Model for effects of supernumerary crossovers in *C*. *elegans* meiosis.

One protein that we hypothesized might contribute to error correction is LEM-3, a late-acting nuclease proposed to resolve chromatin connections in mitotic and meiotic anaphase but that is not thought to affect either crossover numbers or chromosome segregation in the absence of persistent recombination intermediates [33,34]. Therefore, we depleted *lem-3* with RNAi in the *meT7* strain and assessed chromosome segregation. In support of our hypothesis, we found that *lem-3*-depleted *meT7* oocytes had higher incidences of bridges and segregation defects. Specifically, there was a modestly higher proportion of anaphase I bridges at 25°C (25/49, 51.0%, in control vs. 27/37, 72.97%, in *lem-3 RNAi*; P = 0.047, Fisher’s exact test, two-tailed; Fig 7A). Importantly, these defects persisted into Meiosis II in higher proportions compared to oocytes without *lem-3* depletion, with MII spindles having significantly increased instances of chromosome segregation issues (categorized as having either tethered polar bodies or aneuploidy) at 25°C, the temperature that causes increased CO events along *meT7* (15/41, 36.6% in control vs. 20/28, 71.4% in *lem-3 RNAi*; P = 0.007, Fisher’s exact test, two-tailed; Fig 7B). Live imaging of *lem-3* depleted *meT7* oocytes corroborated these findings, showing an increase in chromosome segregation defects, including two cases in which chromatin bridges appeared to fragment (2/13, 15%), and one failure to extrude a Meiosis I polar body (1/13, 7%) (Fig 7D; S1 Table). In five cases, the *meT7* chromosome was unable to segregate in anaphase I and was subsequently extruded into the first polar body (5/13, 38%) (Fig 7D). Further, we saw a 6.3% increase in embryonic lethality in *meT7* treated with *lem-3* RNAi (27.6% *meT7* untreated vs. 33.9% *meT7 + lem-3* RNAi; P = 0.0008; Fisher’s exact test, two-tailed; Table 1) that is a larger increase than the 0.9% that is observed in N2 treated with *lem-3* RNAi (0.5% N2 untreated vs. 1.4% N2 + *lem-3* RNAi; P = 0.02; Fisher’s exact test, two-tailed; Table 1). Together, these results suggest that LEM-3 plays a role in correcting *meT7* chromosome segregation defects during oocyte meiosis.

Furthermore, our analysis suggested another potential mechanism that could contribute to error correction in cases of chromatin bridges caused by excess COs. Specifically, we noticed that when oocytes had a chromosome tethered to the polar body that persisted into anaphase II, the segregating sister chromatid connected to the polar body via a chromosome tether was located closer to the cortical side (23/26 cases; 3 examples shown in Fig 7C), suggesting that the sister chromatid associated with the chromosome tether may be expelled with the second polar body, leaving the oocyte with the untethered sister chromatid and therefore euploid (*i*.*e*. with the correct number of chromosomes). This fixed imaging result is supported by our live imaging, where we found that in 4/5 movies containing persistent chromosome tethers in anaphase II (Fig 6A v-vi and 6B v-vi), the sister chromatid associated with the chromosome tether was extruded with the second polar body during Meiosis II (Fig 6A v and 6B v). Currently, it is unclear whether the oocyte actively recognizes the sister chromatid with the chromosome tether or whether the chromosome tether biases the direction of spindle rotation towards the cortex, thereby facilitating elimination of tethered sister chromatids by their deposition into the polar body. In either case, this mechanism could serve to eliminate improperly segregated chromosomes from the resulting gamete.

## Discussion

In summary, our work provides molecular insights into how crossover limitation promotes proper chromosome congression and segregation in the oocyte. We found that increased COs cause defects in bivalent organization that likely impact the ability of chromosomes to achieve metaphase alignment (Fig 7E). In normal meiosis, bivalents organize around a single CO into a structure with long and short arms, and then a ring complex (RC) composed of essential meiotic proteins forms around the short arm interface. In contrast, an increased number of COs leads to the designation of additional short arms and subsequent formation of extra RCs. Interestingly, when multiple RCs form along a single chromosome, they all appear to be functional. The inability to inactivate extra RCs in *C*. *elegans* meiosis appears to have consequences, as there are chromosome congression errors that may arise from differential forces exerted on bivalents. This result highlights the importance of proper RC patterning, and demonstrates that the structure of this protein complex affects its function. Moreover, our studies revealed that increasing CO number has consequences for homolog segregation, as bivalents with excess crossovers have frequent chromatin bridging during the first meiotic division (Fig 7E).

### Crossover limitation promote accurate chromosome segregation

Strong CO interference, where there is only ~1–3 COs per chromosome pair per meiosis, is observed in several model systems, including fruit flies, worms, and mice [1]. Even in human oocytes, CO interference is strong, with 1–2 COs per homolog [35]. Although CO interference was initially observed over a century ago in *Drosophila* [2,3] and was subsequently found to be widely conserved among many model systems [1], the consequences of extra interfering COs along a pair of homologous chromosomes has not been directly determined. Our study is the first, to our knowledge, in any organism to assess these consequences. Our findings using fusion chromosomes in *C*. *elegans* suggest that CO interference helps promote proper meiotic chromosome alignment by limiting homolog pairs to one crossover per bivalent under normal circumstances (thereby preventing the difficulties that ensue when bivalents assemble multiple RCs). Moreover, we also observed severe chromosome segregation defects in the presence of extra COs. These segregation errors could have multiple causes. First, the inability to properly congress chromosomes could be one contributing factor; if anaphase is initiated when the *meT7* bivalent is not aligned, this could cause errors. However, the defective bivalent structure that arises in the presence of extra COs likely also causes other problems, beyond the RC defects. Second, there could be defects resolving recombination intermediates when extra COs are present, thereby resulting in the thick anaphase I bridges that we observe in our study. Another possibility is that limiting COs could promote the creation of a chromosome structure that facilitates the loss of cohesion in the correct domain. Since AIR-2 is thought to phosphorylate the cohesin subunit REC-8 to enable cleavage by separase [11,36], the AIR-2 mispatterning that occurs in the presence of extra crossovers could lead to defects in cohesion release, which may cause chromosome bridging. Finally, it is also possible that when a bivalent has multiple crossovers, this may involve 3–4 chromatids (instead of the usual two chromatids in a single crossover), which may result in chromosome bridging and segregation issues.

Overall, the ability to limit crossovers is important for the faithful execution of meiosis, which may represent a driving force that has contributed to the conservation of CO interference in many organisms. In the future, it will be interesting to investigate how supernumary COs affect meiotic chromosome segregation in other organisms. This is important since *C*. *elegans* oocytes have a number of features not shared by all organisms, including holocentric kinetochores [37], reliance on the RCs for chromosome congression [12,19], and a unique chromosome segregation mechanism [13]. Given the conservation of CO interference, we predict that excess COs may also affect the fidelity of chromosome segregation in other organisms, but this remains to be experimentally determined. Moreover, it will also be important to investigate this question using normal length chromosomes, as it is possible that some the defects we documented for *meT7* and *mnT12* may not be exclusively caused by excess COs, and that other properties of the fused chromsomes may contribute.

### Crossover interference and holocentric chromosomes

In comparison to humans and other model organisms, *C*. *elegans* exhibits extremely strong CO interference known as complete CO interference, where one (and only one) CO is formed per chromosome pair [4]. One potential reason why CO interference may be so robust in *C*. *elegans* is that they have holocentric chromosomes, which assemble centromere and kinetochore proteins along their entire length. In comparison to monocentric organisms, most holocentric organisms experience complete CO interference, suggesting that there is a strong selection bias for this highly stringent form of CO interference in these organisms [38,39].

In contrast to monocentric chromosomes, holocentric chromosomes are faced with the unique challenge that microtubules could theoretically attach to any part of the bivalent surface, rather than associating in a manner that promotes the biorientation of homologous chromosomes [40]. One way that *C*. *elegans* counteract this problem is the asymmetric positioning of a single crossover along the length of a chromosome; this generates a single point of organization along the length of each chromosome, which patterns the chromosome in a manner that facilitates chromosome biorientation, alignment, release of cohesion, and segregation [40,41]. Moreover, a recent *C*. *elegans* study has found that the asymmentric placement of the crossover along the chromosome is important for proper chromosome remodeling and segregation [42]. Perhaps the acquisition of holocentric chromosomes requires extreme crossover interference in addition to asymmetric placement of a crossover along the chromosome length. Future experiments or analysis of the evolution of holocentric organisms may parse out the correlation between holocentricity and strong crossover interference.

### Mechanisms exist to correct errors caused by supernumary crossovers

Our studies also uncovered two mechanisms that counteract defects caused by excess COs and likely account for some of the relatively low embryonic inviability in fusion chromosome strains. We found that depletion of the conserved nuclease LEM-3/Ankle1 increases the frequency and persistence of anaphase bridging, consistent with its previously proposed role in processing erroneous recombination intermediates and correcting meiotic errors [34]. Given the role of LEM-3 in processing anaphase bridges from persistent recombination intermediates, it is possible that some of the chromatin bridges we are observing from excess COs along *meT7* are unresolved crossover recombination intermediates. In the future, it will be interesting to determine whether additional proteins assist in processing DNA tethers. Given the relatively low embryonic lethality of the *meT7* fusion chromosome strain in contrast to the frequency of DNA tethers and other segregation errors, it is likely that other correction mechanisms exist. In addition to its role in meiotic recombination, the BLM/HIM-6 helicase (which localizes to interfering CO sites [43]) has been shown in multiple systems to localize to and to help resolve DNA bridges during both mitotic and meiotic chromosome segregation [44–49]. Similarly, the nucleases MUS-81, SLX-1 and SLX-4 have genetic interactions with both LEM-3 [34] and HIM-6 [50] and may act combinatorially to resolve DNA bridging due to excess COs. Further, topoisomerase II has been shown to remove DNA tethers from heterochromatin regions that connect achiasmate homologs in *Drosophila* [51]. Given the conservation of the nuclease LEM-3/Ankle1, these mechanisms that counteract the effects of multiple COs may be augmented in organisms that occasionally experience more than one CO along a chromosome.

Although nucleases can serve as a generalized solution to resolve erroneous DNA connections, we also discovered a second mechanism specific to oocytes. Since each CO involves only two of the four sister chromatids, there could be cases where one sister chromatid of *meT7* is tethered to the polar body due to the presence of an unresolved chiasma, but the other sister chromatid is unaffected since it was not involved in the CO that caused the problem. Thus, if the tethered sister chromatid is expelled from the oocyte in anaphase II and the normal chromatid is retained, the egg would be euploid. We found that in oocytes where a chromosome tether was not resolved in anaphase I, the tethered sister chromatid was preferentially extruded from the oocyte in anaphase II, revealing another mode of error correction. This mechanism is made possible by the asymmetric nature of the oocyte divisions, in which half of the genetic material is discarded into the polar body during each division. This asymmetry has been shown to allow the preferential elimination of univalent chromosomes in *C*. *elegans* [52], and here may provide a second opportunity for correction of errors associated with excess COs. It is interesting to speculate on whether the oocyte spindle actively recognizes the chromosome tether, or whether the chromosome tether passively biases the rotation of the spindle, positioning the tethered sister chromatid adjacent to the cortex, where it will be extruded into the second polar body. Currently, little is known about how spindle rotation is influenced in *C*. *elegans*; however, previous work has shown that the minus-end binding protein ASPM-1 is brighter on the spindle pole that rotates towards the cortex, suggesting that spindle asymmetry could play a role [53] similar to spindle asymmetry and selfish centromeres in mouse oocytes [54]. Going forward, it would be interesting to ask whether unresolved DNA connections influence spindle factors to allow for preferential spindle rotation as a form of meiotic drive.

### Licensing of Ring Complex formation by interfering crossovers

Our results further establish how the coordination and interplay between recombination and chromosome segregation are critical for the maintenance of genomic integrity through generations. We observed that the occurrence of multiple Class I interfering crossovers leads to the formation of multiple RCs. Moreover, we found a strong correlation between interfering CO number and eventual RC number along a single chromosome. This result leads to the compelling hypothesis that an interfering CO site may be a licensing event for RC formation. Through either its specific DNA conformation or the recombination machinery associated with it, a CO may designate a location along the chromosome for RC components to assemble. Previous experiments have already found that Aurora B kinase (AIR-2) localizes to sites of COs [8]. Further, the HIM-6/BLM helicase has been found to localize to interfering CO sites and help maintain chiasmata until metaphase I [47,49]. Future experiments exploring whether there is direct interaction between recombination machinery and RC components may establish this connection between CO sites and RC formation.

Our study finds that excess Class I interfering COs affect chromosome segregation in *C*. *elegans*. In contrast, the occurrence of excess Class II non-interfering crossovers does not affect meiotic chromosome segregation in *Arabidopsis thaliana*, *Schizosaccharomyces pombe*, and *Aspergilis nidulans* [55–58]. *C*. *elegans* normally do not form Class II non-interfering COs, but in a mutant for the helicase *RTEL-1*, non-interfering COs are now formed alongside interfering COs [59]. Notably, in *rtel-1* mutants, this 16-fold increase in non-interfering COs results in only a very modest effect on embryonic lethality (3%; [60]), suggesting that an excess of non-interfering COs does not affect chromosome segregation. The differences between the effects of excess Class I versus Class II COs on chromosome segregation suggests the possibility that interfering COs are differentiated from non-interfering COs at the level of licensing a location for RC formation.

Overall, our study provides new molecular insights into how excess interfering COs affect chromosome congression and segregation. Further, our results indicate the existence of mechanisms to assist with correcting errors associated with the formation of excess COs, thereby lending important insight into how some organisms, such as *Saccharomyces cerevisiae*, can tolerate more than two interfering COs per chromosome. Future studies investigating why certain organisms can experience higher levels of interfering COs will lend further insight into these critical mechanisms for maintaining genomic integrity through generations.

## Materials and methods

### *C*. *elegans* strains, genetics, and culture conditions

All strains are from the Bristol N2 background and were maintained at 15°C or 20°C and crossed at 20°C under standard conditions. Temperatures used for specific experiments are indicated below. For all experiments with meiotic mutants, homozygous mutant worms were derived from balanced heterozygous parents by selecting progeny lacking a dominant marker (Unc and/or GFP) associated with the balancer. For experiments marked 25°C, L4 worms were shifted to 25°C 24–48 hours prior to dissection. For lower temperature controls, either 15°C or 20°C was used; these temperatures are both well below the identified threshold for destabilization of the synaptonemal complex (≥25°C; [61]), which has a role in crossover number and formation [5,62].

The following strains were used in this study:

N2: Bristol wild-type strain. [Caenorhabditis Genetics Center (CGC)]
SP646: *mnT12 (IV;X)*. [Caenorhabditis Genetics Center (CGC)]
AV311: *dpy-18(e364) unc-3(E151) meT7*(*III; X; IV*). [7]
AV630: *meIs8[unc-119(+) Ppie-1*::*gfp*::*cosa-1] II*. [6]
AV695: *meIs8[unc-119(+) Ppie-1*::*gfp*::*cosa-1] II*; *mnT12 (IV;X)*. [5,6]
OD868: *ItSi220*[pOD1249/pSW077; *Pmex-5*::*GFP-tbb-2-operon-linker-mCherry-his-11; cb-unc119(+)*] *I*. [63]
DLW11: *meIs8[unc-119(+) Ppie-1*::*gfp*::*cosa-1] II*; *dpy-18(e364) unc-3(e151) meT7* (*III;X;IV*). (This study)
DLW30: *ItSi220 I*; *dpy-18(e364) unc-3(E151) meT7*(*III; X; IV*). (This study)

### RNAi treatments

RNAi by feeding was performed as previously described [19]. Worms were synchronized at the L1 phase by bleaching adults and allowing resultant eggs to hatch on unseeded NGM plates at 20°C for 20–24 hrs. Synchronized L1s were then washed off of the unseeded NGM plates with M9 and placed on NGM+1mM IPTG+100μg ampicillin plates that were poured within 30 days of use and freshly seeded one day before use with clones picked from the Ahringer Lab RNAi feeding library [64] or the empty L4440 vector (referred to as “control RNAi” in figures and text). The RNAi plates with L1s were then placed at 15°C and grown to adulthood. For RNAi experiments performed at 25°C, L4 worms were shifted to 25°C for 40–48 hours prior to dissection.

### Immunofluorescence for late meiotic prophase I

Immunofluorescence was performed as in [5]. Gonads from adult worms at 18–24 hours post-L4 stage were dissected in 1x egg buffer with 0.1% Tween on VWR Superfrost Plus slides, fixed for 5 min in 1% paraformaldehyde, flash frozen with liquid nitrogen, and then fixed for 1 minute in 100% methanol at -20°C. Slides were washed 3 x 5 min in 1x PBST and blocked for one hour in 0.7% BSA in 1x PBST. Primary antibody dilutions were made in 1x PBST and added to slides. Slides were covered with a parafilm coverslip and incubated in a humid chamber overnight (14–18 hrs). Slides were washed 3 x 10 min in 1x PBST. Secondary antibody dilutions were made at 1:200 in 1x PBST using Invitrogen goat or donkey AlexaFluor labeled antibodies and added to slides. Slides were covered with a parafilm coverslip and placed in a humid chamber in the dark for 2 hrs. Slides were washed 3 x 10 min in 1x PBST in the dark. All washes and incubations were performed at room temperature, unless otherwise noted. 2 μg/ml DAPI was added to slides and slides were subsequently incubated in the dark with a parafilm coverslip in a humid chamber. Slides were washed once for 5 min in 1x PBST prior to mounting with Vectashield and a 20 x 40 mm coverslip with a 170 ± 5 μm thickness. Slides were sealed with nail polish immediately following mounting and then stored at 4°C prior to imaging. All slides were imaged (as described below) within two weeks of preparation. The following primary antibody dilutions were used: rabbit anti-GFP (1:1000) [6]; chicken anti-GFP (1:1000) (Abcam 13970); guinea pig anti-SYP-1 (1:200) [62]; rabbit anti-HIM-3 (1:200) [65]; chicken anti-HTP-3 (1:500) [66]; rabbit HTP-1/2 (1,500) [8].

### Immunofluorescence for prometa-, meta- and anaphase I and II

Immunofluorescence was performed as in [67]. Briefly, adult worms were picked into a drop of M9 media on poly-l-lysine coated slides (Fisher Scientific), cut in half to release oocytes, covered with a coverslip and frozen for 7 minutes in liquid nitrogen. The coverslip was quickly cracked off, and slides were fixed in -20°C methanol for 35 minutes. Samples were then rehydrated in PBS and blocked in AbDil (PBS with 4% BSA, 0.1% Triton X-100, 0.02% Sodium Azide) for one hour at room temperature, and then washed with PBST (PBS + 0.1% Triton X-100). Primary antibodies were diluted in AbDil, applied to samples and incubated overnight at 4°C. Samples were washed with PBST, and secondary antibodies were diluted in AbDil, applied to samples, and incubated for 2 hours at room temperature. Slides were washed and incubated with Hoechst 33342 (Invitrogen) at 1:1000 in PBST for 10 minutes at room temperature. Slides were washed a final time and mounted in 0.5% p-phenylenediamine in 90% glycerol, 20mM Tris, pH 8.8, sealed with nail polish, and stored at 4°C prior to imaging.

The following antibodies were used: rat anti-AIR-2 (1:500) [25], rabbit anti-SEP-1 (1:200, gift from Andy Golden), rabbit anti-BIR-1 (1:800, this study), mouse anti-SUMO (1:500, gift from Federico Pelisch), rabbit anti-BUB-1 (1:1000) [17], rabbit anti-ASPM-1 (1:5000, gift from Arshad Desai), Mouse anti-α-tubulin-FITC (1:500, DM1α, Sigma) and rabbit anti-KLP-19 (1:2500, this study). KLP-19 and BIR-1 polyclonal antibodies were generated by Covance using recombinant GST-BIR-1 (Full length protein) and GST-KLP-19 (amino acids 371–1084) as antigens (purification performed as in [17]). Antibody sera was then affinity purified and used at indicated concentrations. Alexa-fluor conjugated secondary antibodies were used at 1:500.

### Fixed imaging

For Fig 1, S1 Fig, S2 Fig and S3 Fig, IF slides were imaged at 512 x 512 pixel dimensions on an Applied Precision DeltaVision microscope using a 60x objective (NA = 1.42) with 1.5x optivar. Images were acquired as Z-stacks at 0.2 μm intervals and deconvolved with Applied Precision softWoRx deconvolution software. For quantification of GFP::COSA-1 foci, nuclei that were in the last 4–5 rows of late pachytene and were completely contained within the image stack were analyzed. Foci were quantified manually from deconvolved three-dimensional stacks. *meT7* chromosomes in pachytene nuclei were identified based on size. Regardless of temperature (20°C or 25°C), all three wild-type/non-fused chromosomes in the nuclei of the *meT7* strains contained only one GFP::COSA-1 focus per chromosome (n = 30), which is consistent with all chromosomes in wild-type strains and the wild-type/unfused chromosomes in *mnT12 (X;IV)* fusion chromosome strain (this study, [5,6]). Given the consistency of COSA-1 counts for the 3 wild-type/non-fused chromosomes in the *meT7* strains, the number of COSA-1 foci per *meT7* was calculated by subtracting 3 from the total number of COSA-1 foci per nucleus. For visualization and quantitation of chiasmata (Fig 1D), individual *meT7* bivalents from diakinesis nuclei in -2, -3, or -4 oocytes were identified based on size, cropped, and rotated in three-dimensions using Imaris (Bitplane/Oxford Instruments) three-dimensional rendering software. Scoring of chiasmata was based primarily on HTP-3 (chromosome axis) or HIM-3 (chromosome axis) and DAPI staining, as GFP::COSA-1 dissociates from chromosomes during progression through the diakinesis stage. For quantification of DAPI body counts during diakinesis, counts were performed as described in [6]. For Fig 1B and 1C, images shown are projections through three-dimensional data stacks encompassing whole nuclei, generated with a maximum-intensity algorithm with the softWoRx (Applied Precision) software. For Fig 1D and S3 Fig, the images of *meT7* and *mnT12* bivalents shown are snapshots of an Imaris three-dimensional rendering of individual diakinesis bivalents with maximum intensity rendering for HTP-3 or HTP-1/2 with SYP-1. The images of wild type unfused autosome bivalents in Fig 1D are projections through three-dimensional data stacks encompassing the whole bivalent, generated with a maximum intensity algorithm with the softWoRx (Applied Precision) software.

For Figs 2, 3, 4, 5 and 7A, S5 Fig, S6 Fig and S7 Fig, fixed sample IF slides were imaged at 256 x 256 pixel dimensions on an Applied Precision DeltaVision microscope using a 100x oil objective (NA = 1.4), housed in the Northwestern University Biological Imaging Facility supported by the Northwestern University Office for Research. Images were acquired as Z-stacks at 0.2 μm intervals and deconvolved (ratio method, 15 cycles) with Applied Precision softWoRx deconvolution software. Images are maximum projections of entire spindles unless otherwise noted in the figure legends. Meiosis stages were determined by eye based on protein localization, chromosome-to-chromosome distance, chromosome size and polar body presence. *meT7* chromosomes were identified based on size.

### Ring stretching assay

Ring stretching on monopolar spindles in Fig 3 was performed as in [19]. Worms were grown on RNAi plates with a 1:1 mixture of *emb-30* and *klp-18* RNAi feeding clones from the Ahringer library to induce an arrest in metaphase on a monopolar spindle. RC components tend to stretch away from bivalents in extended metaphase arrest; spindles in extended arrest were determined by eye based on significant ring stretching of the three normal bivalents. The number of stretching rings on either *meT7* or a control normal bivalent was counted using Imaris.

### Image quantification

#### Ring structure quantification

To assess ring structure, RCs on *meT7* or *mnT12* bivalents were rotated in Imaris; prometaphase and metaphase oocytes with bipolar spindles were analyzed. Rings were scored by eye and considered mispatterned if they had more than one plane, consisted of more than one individual unit, or had more than one major loop. For Fig 2A and S5A Fig, rings were considered “slightly mispatterned” if they consisted of two distinct units on a single plane or appeared as two connected loops on a single plane; given the resolution of our images, it was not possible to discern in most cases if this represents one ring with an aberrant shape, or two distinct rings in the same plane. Rings with more than two units, multiple loops at different angles or planes on the bivalent, or rings with many fragments were considered “severely mispatterned.” For Fig 2E, quantification of ring structure was performed in MII, and the *meT7* fusion chromosome was identified by its larger size. Because our analysis indicates that MI chromosome segregation is sometimes aberrant (Figs 5 and 6), it is possible that some of the analyzed MII *meT7* chromosomes had not been fully segregated in MI; this could potentially account for some of the MII ring structure defects observed.

#### Linescans

For Fig 2, linescans were performed in ImageJ. Fluorescence intensity linescans of normal or fused chromosomes were performed along the pole-pole axis, as determined by tubulin intensity, at 40 x 30 pixels (L x W) (n = 25). Only clearly bipolar spindles were used for analysis, and all images had the same exposure conditions. Both chromosome length and fluorescence intensity were normalized to a maximum of 1, and the average (solid line) and standard error of the mean (SEM, shaded), of both DNA and SUMO were plotted using the ggplot2 package in Jupyter Notebook.

#### Metaphase alignment quantification

For Fig 4A, *meT7* worms were arrested in metaphase using *emb-30* RNAi. The metaphase plate was determined by rotating the image in 3D using Imaris until a single plane could be determined by eye based on SUMO-stained RCs on chromosomes I, II and V. The average total width of SUMO intensity of chromosomes I, II and V was measured using Imaris per spindle at the determined metaphase plate. The *meT7* fusion chromosome was considered “unaligned” if greater than 50% of its SUMO intensity fell outside of 2 standard deviations of the average range of SUMO on chromosomes I, II and V from the determined metaphase plate.

#### Chromosome distance measurements

For Fig 4B, chromosome-to-pole distances were measured using Imaris. The center of the monopole was determined by using the “Surfaces” tool to determine the volume of the ASPM-1 region and to assign the center of this volume. Then, the distance between this point and the center of each chromosome was measured. Per spindle, the average of the distances to chromosomes I, II and V was subtracted from each individual distance to chromosomes I, II, or V (green points on the diagram/graph in Fig 4C) or to the fused chromosome (blue points).

#### Bivalent roundness quantification

For S7C Fig, bivalent roundness was determined by dividing the length of the shortest axis of an individual bivalent by the length of the longest axis. Sum projections of 3-slice fixed IF images were created in ImageJ, and the DNA channel was thresholded to define the bivalents. An oval shape was fit to these thresholded images, and the “circularity” of each bivalent was calculated using the “Analyze Particles” tool. The data were plotted in Jupyter Notebook.

#### Chromatin bridging quantification

For Figs 5, 6 and 7, and S5 Fig, DNA was denoted as bridged if in anaphase the width of the bridge was less than the width at either end of the segregating chromosomes. Otherwise, a single *meT7* or *mnT12* chromosome mass in anaphase was marked as showing delayed segregation, and two distinct segregating *meT7* or *mnT12* masses with no connecting DNA were considered wild type.

#### Meiosis II quatification

For Figs 5 and 7 and S5 Fig, ploidy was determined by eye based on chromosome counts on MII spindles (*i*.*e*. oocytes were considered aneuploid if the number of Hoechst-stained bodies was not equal to 4). A polar body tether was defined by a clear continual strand of DNA connecting a polar body to a chromatid pair within a forming or formed MII spindle. Polar bodies were determined to be DNA masses in close proximity to, yet remaining outside of, forming MII spindles.

### Scoring embryonic viability

Viability counts (percent hatching) were determined by singling out 5–15 L4s onto individual plates and growing them at the temperatures indicated in the figures/tables until broods were produced. Mothers were transferred to new plates each day and allowed to produce broods until they no longer laid fertilized embryos. After mothers were moved, embryos and larvae were counted, and then returned to 25°C and allowed to develop for 18–24 hours (wild type) or 40–48 hours (*meT7*) before counting unhatched embryos. *meT7* embryos were given more time to develop, as the strain develops more slowly than wild type.

### Live imaging

Live imaging of oocyte meiotic chromosome segregation in wild type and *meT7* fluorescent fusion lines was accomplished by cutting open adult worms with a single row or less of embryos in 4μl of egg buffer (118mM NaCl, 48mM KCl, 2mM CaCl_2_, 2mM MgCl_2_, and 0.025 mM HEPES, filter sterilized before HEPES addition) on a coverslip and gently mounting onto a 2% agarose pad on a microscope slide. Worms were synchronized by hypochlorite hatch-off, and grown at 20°C; worms were then upshifted to 25°C overnight (16–20 hours) before imaging. Oocytes were imaged using a spinning disk confocal unit, CSU-W with Borealis (Andor), and dual iXon Ultra 897 (Andor) cameras mounted on an inverted Leica DMi8 microscope, with a 100x HCX PL APO 1.4NA oil objective lens (Leica). The imaging system was controlled via Metamorph (Molecular Devices) software. Oocytes were imaged every 5 seconds with 1μm Z-spacing (16μm total Z-stack) and the 488nm and 561nm channels were imaged simultaneously. After recording, movies were maximum projected, cropped, and color channels were adjusted independently for brightness and contrast in ImageJ (National Institutes of Health).

### Statistical analysis

All reported P-values and statistical tests are specified in the text and figure legends. A P-value less than 0.05 was considered statistically significant.

## Supporting information

S1 Fig Quantification of DAPI bodies at diakinesis in wild type (N2), *meT7*, and *mnT12* oocytes. Representative diakinesis of each genotype and the quantification of the average number of DAPI bodies at diakinesis for wild type (6.0±0; N = 22 nuclei), *meT7* (4.0±0.2; N = 28 nuclei), and *mnT12* (5.0±0.2; N = 21 nuclei) fixed nuclei. Error bars indicate standard deviation. Scale bars = 5μm.
(TIF)

S2 Fig Quantification of GFP::COSA-1 on unfused wild type chromosomes and the *mnT12* fusion chromosome. Quantification of the average number of GFP::COSA-1 in immunofluorescence images of fixed unfused wild type chromosomes (from AV630) and the *mnT12* fusion chromosome (from AV695) at 20°C (dark blue) and 25°C (light blue). Unfused wild type chromosomes display essentially only one COSA-1 focus per chromosome at either temperature (20°C N = 3030; 25°C N = 486). The *mnT12* fusion chromosome has either one or two COSA-1 foci and increasing the temperature to 25°C causes an increase in the number of *mnT12* chromosomes with two COSA-1 foci (20°C N = 150; 25°C N = 85).
(TIF)

S3 Fig HTP-1/2 and SYP-1 patterning on diakinesis chromosomes. Representative immunofluorescence images of fixed diakinesis chromosomes stained with HTP-1/2 (magenta) and SYP-1 (yellow) from an unfused autosome, the *meT7* fusion chromosome, and the *mnT12* fusion chromosome. The unfused autosome is from an *mnT12* nucleus and displays normal patterning of HTP-1/2 on the long arm and SYP-1 on the short arm. In contrast, both fusion chromosomes display defects in establishing the long arm and short arm patterning of HTP-1/2 and SYP-1. Dashed lines indicate traced SYP-1 (yellow) and HTP-1/2 (magenta) along the chromosome axes. Scale bars = 1μm.
(TIF)

S4 Fig Models for chromosome organization in *meT7* bivalents. Cartoon images show potential models for how short arm interfaces are distributed on *meT7* prometaphase bivalents, and how they may relate to different ring complex (RC) patterns from fixed oocyte zooms in Fig 2A. One whole ring (second row) may result from a single short arm interface, recruiting RC components in a manner similar to a wild-type-size bivalent (top row) on prometaphase bivalents. *meT7* bivalents with a slight mispatterning RC (third row) may have two short arm interfaces, one at each end of the paired homologs, leading to a structure with ring complex components that are distinct, yet visible on the same plane. For bivalents with more complex RC mispatterning (bottom row), it is likely that the short arm interface is significantly impaired, potentially due to incorrect crossover number, and the targeted RC is not restricted to a single plane or a coherent, ordered structure in prometaphase.
(TIF)

S5 Fig *mnT12* oocytes show similar phenotypes to *meT7* oocytes. (A) Immunofluorescence and quantification of AIR-2 localization in fixed *mnT12* oocytes. Single-bivalent zooms of *mnT12*, indicated by arrows, show that we see similar categories of AIR-2 localization as *meT7*: whole ring (top row), slight mispatterning (middle row), or severe mispatterning. 0% (0/40 at 15°C and 0/35 at 25°C) of normal bivalents showed mispatterned AIR-2 localization in *mnT12* oocytes. At 15°C, *mnT12* bivalents were slightly mispatterned in 8/36 oocytes and severely mispatterned in 1/36 oocytes, while at 25°C *mnT12* bivalents were slightly mispatterned in 9/27 oocytes and severely mispatterned in 3/27 oocytes. (B) Chromatin bridges, indicated by arrows, are present in fixed *mnT12* anaphase oocytes. In N2 mid-to-late anaphase oocytes, 0/75 spindles contained anaphase bridging. However, 3/18 *mnT12* spindles showed chromatin bridges at 15°C, and 3/11 *mnT12* anaphase spindles showed chromatin bridges at 25°C. (C) Fixed *mnT12* oocytes show persisting consequences of anaphase bridging in Meiosis II. No chromatin-tethered polar bodies or anueploid Meiosis II spindles were observed in N2 Meiosis II oocytes. At 15°C, 3/25 Meiosis II *mnT12* oocytes contained DNA tethered to the polar body, and 1/25 Meiosis II *mnT12* oocytes was aneuploid. At 25°C, 6/27 Meiosis II *mnT12* oocytes had tethered polar bodies, and 3/27 of Meiosis II *mnT12* oocytes were aneuploid. All scale bars = 2.5μm.
(TIF)

S6 Fig Additional characterization of CPC components (related to Fig 2). Immunofluorescence showing that BIR-1 (green) and AIR-2 (red) colocalize on all ring structure types on *meT7* in fixed oocytes. Scale bars = 2.5μm.
(TIF)

S7 Fig Kinetochore proteins localize to misshapen *meT7* bivalents. (A) Immunofluorescence of BUB-1 in prometaphase *meT7* oocytes. BUB-1 targets to the entirety of meiotic bivalents in *meT7*, shown in a full projection (top row), and specifically cups holocentric normal bivalents (bottom row, left) and *meT7* fused bivalents (bottom row, right), as shown in single slices. Scale bars = 2.5μm. (B) Immunofluorescence of SEP-1 in fixed prometaphase *meT7* oocytes. SEP-1 targets to the entirety of meiotic bivalents in *meT7*, shown in a full projection (top row), and specifically cups holocentric normal bivalents (bottom row, left) and *meT7* fused bivalents (bottom row, right), as shown in single slices. Scale bars = 2.5μm. (C) *meT7* bivalents are shaped differently than normal-sized bivalents. Bivalent roundness, or the ratio of the length of the shortest axis on the bivalent to the length of the longest axis, was calculated using ImageJ. This ratio tended to be higher (P = 0.0027, two-tailed Mann-Whitney *U* test) for *meT7* bivalents (N = 51) than normal-sized bivalents (N = 64), suggesting *meT7* bivalents have a less apparent bilobed architecture, and instead have one closer to that of a circle.
(TIF)

S1 Movie Example movie showing *meT7* segregation occasionally (5 of 14 movies) results in the first polar body being tethered to the Meiosis II spindle by a chromatin bridge (related to Fig 6 and S1 Table). Microtubules (GFP::TBB-2) shown in magenta and chromosomes (mCherry::HIS-11), shown in green, during both meiotic divisions in live cells. Playback framerate is 25 frames per second.
(AVI)

S2 Movie Example movie showing *meT7* failed to segregate in Meiosis I and segregated with a chromatin bridge in Meiosis II (1 of 14 movies) (related to Fig 6 and S1 Table). Microtubules (GFP::TBB-2) shown in magenta and chromosomes (mCherry::HIS-11) shown in green during both meiotic divisions in live cells. Playback framerate is 25 frames per second.
(AVI)

S3 Movie Example movie showing *meT7* segregating with a chromatin bridge in Meiosis I that appears to resolve before anaphase of Meiosis II (3 of 14 movies) (related to Fig 6 and S1 Table). Microtubules (GFP::TBB-2) shown in magenta and chromosomes (mCherry::HIS-11) shown in green during both meiotic divisions in live cells. Playback framerate is 25 frames per second.
(AVI)

S1 Table Frequency of chromosome segregation phenotypes from live imaging experiments. (DOCX)
