# Supplementary figures and images for: Excess crossovers impede faithful meiotic chromosome segregation in C. elegans
Source: PLoS Genet. 2020 Sep 4;16(9):e1009001. doi: 10.1371/journal.pgen.1009001 (PMC7508374; doi:10.1371/journal.pgen.1009001)

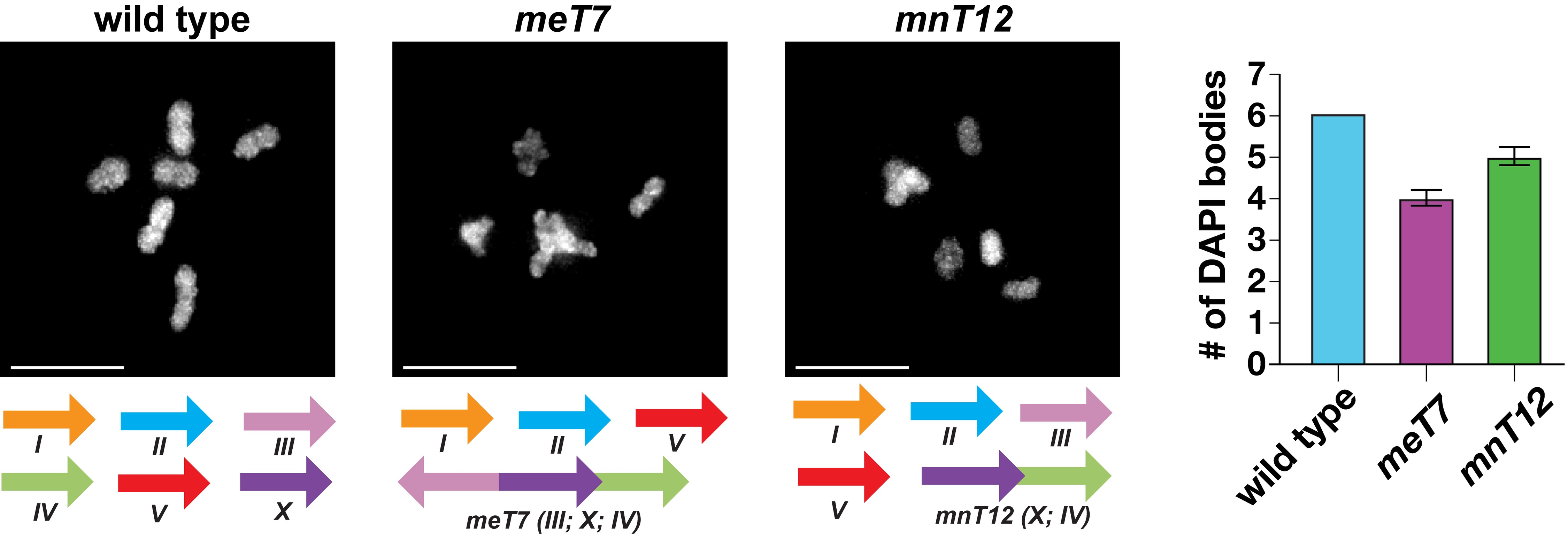

Supplement: S1 Fig — Representative diakinesis of each genotype and the quantification of the average number of DAPI bodies at diakinesis for wild type (6.0±0; N = 22 nuclei), meT7 (4.0±0.2; N = 28 nuclei), and mnT12 (5.0±0.2; N = 21 nuclei) fixed nuclei. Error bars indicate standard deviation. Scale bars = 5μm. (TIF) [file pgen.1009001.s001.tif]

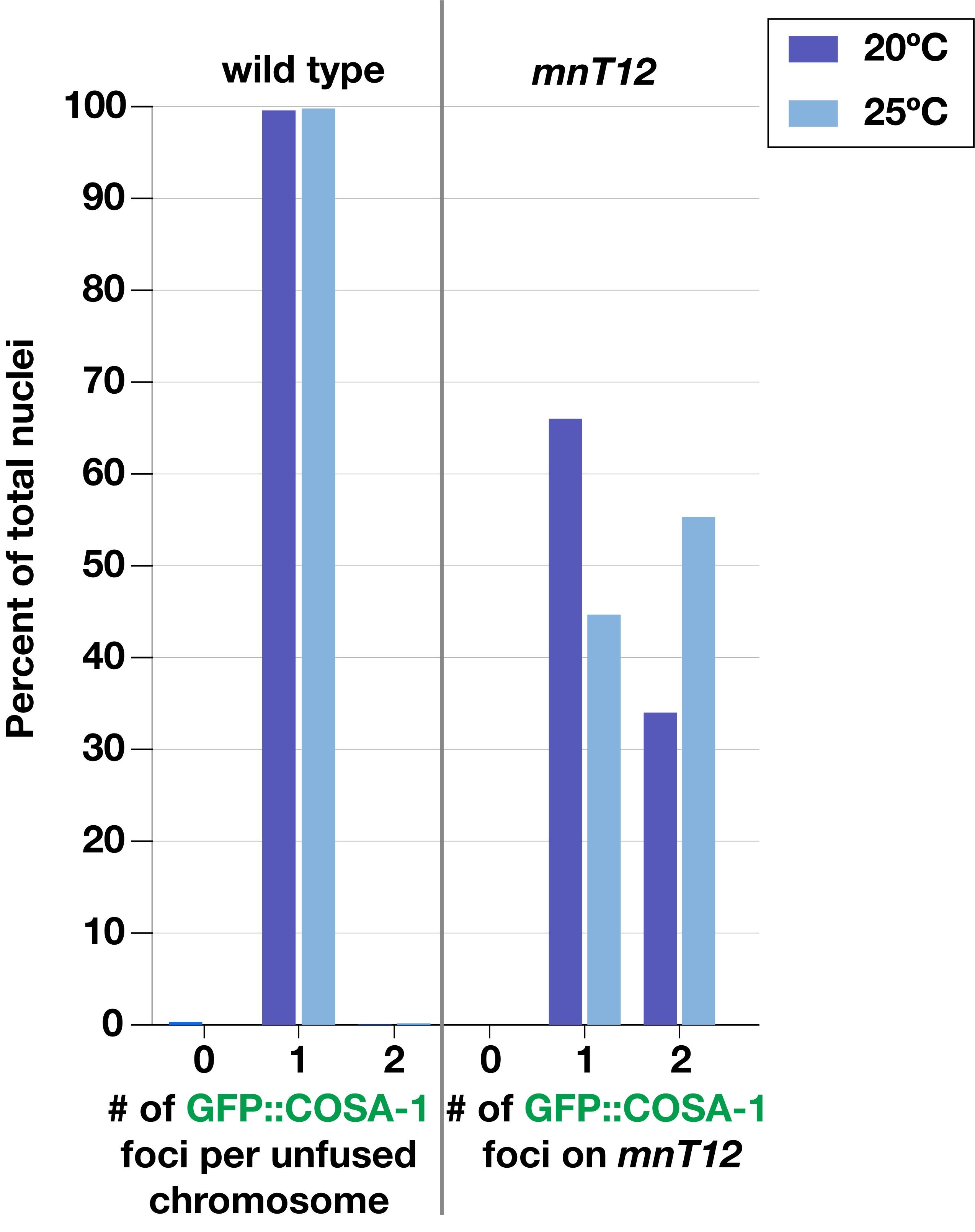

Supplement: S2 Fig — Quantification of the average number of GFP::COSA-1 in immunofluorescence images of fixed unfused wild type chromosomes (from AV630) and the mnT12 fusion chromosome (from AV695) at 20°C (dark blue) and 25°C (light blue). Unfused wild type chromosomes display essentially only one COSA-1 focus per chromosome at either temperature (20°C N = 3030; 25°C N = 486). The mnT12 fusion chromosome has either one or two COSA-1 foci and increasing the temperature to 25°C causes an increase in the number of mnT12 chromosomes with two COSA-1 foci (20°C N = 150; 25°C N = 85). (TIF) [file pgen.1009001.s002.tif]

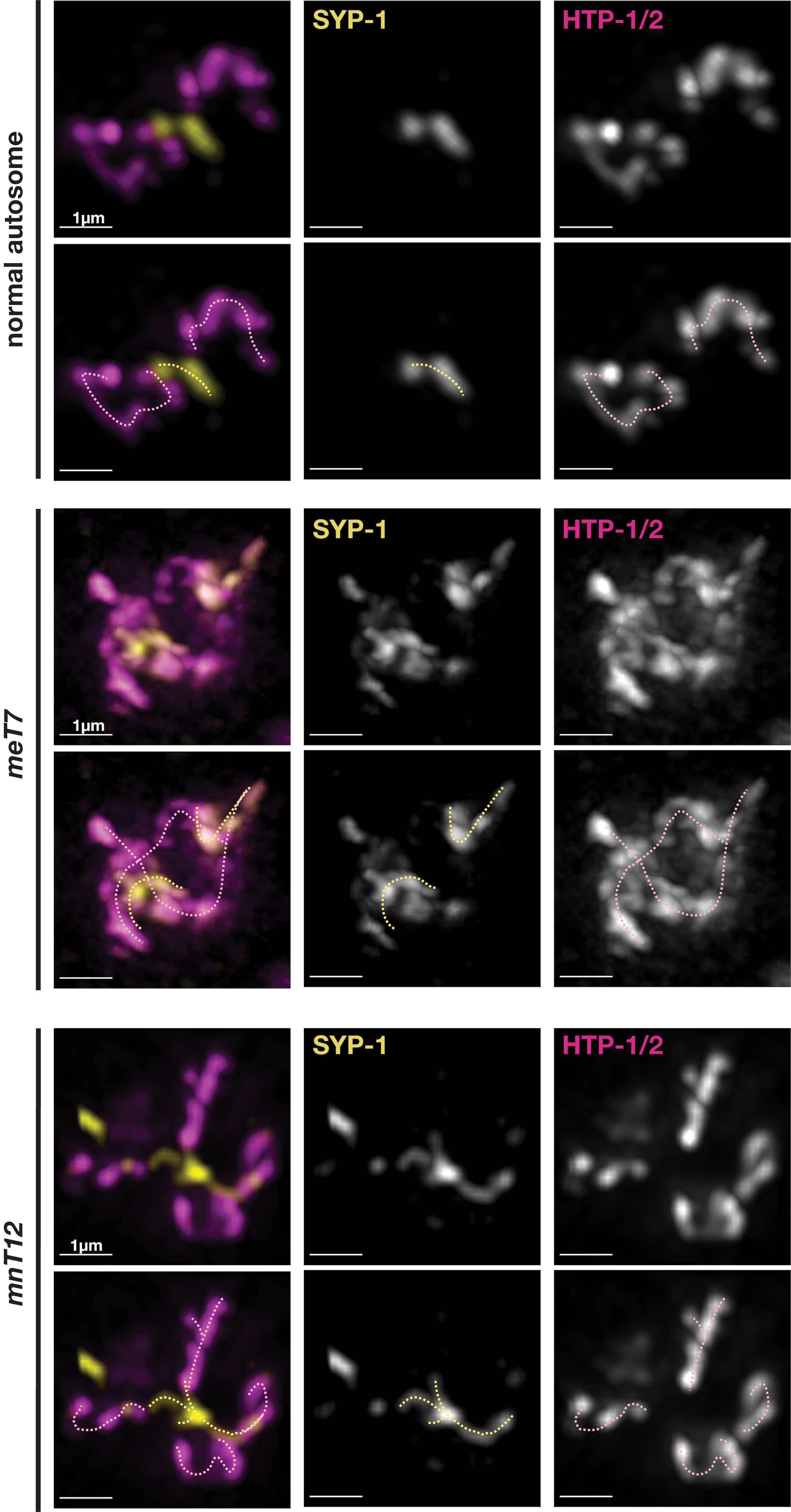

Supplement: S3 Fig — Representative immunofluorescence images of fixed diakinesis chromosomes stained with HTP-1/2 (magenta) and SYP-1 (yellow) from an unfused autosome, the meT7 fusion chromosome, and the mnT12 fusion chromosome. The unfused autosome is from an mnT12 nucleus and displays normal patterning of HTP-1/2 on the long arm and SYP-1 on the short arm. In contrast, both fusion chromosomes display defects in establishing the long arm and short arm patterning of HTP-1/2 and SYP-1. Dashed lines indicate traced SYP-1 (yellow) and HTP-1/2 (magenta) along the chromosome axes. Scale bars = 1μm. (TIF) [file pgen.1009001.s003.tif]

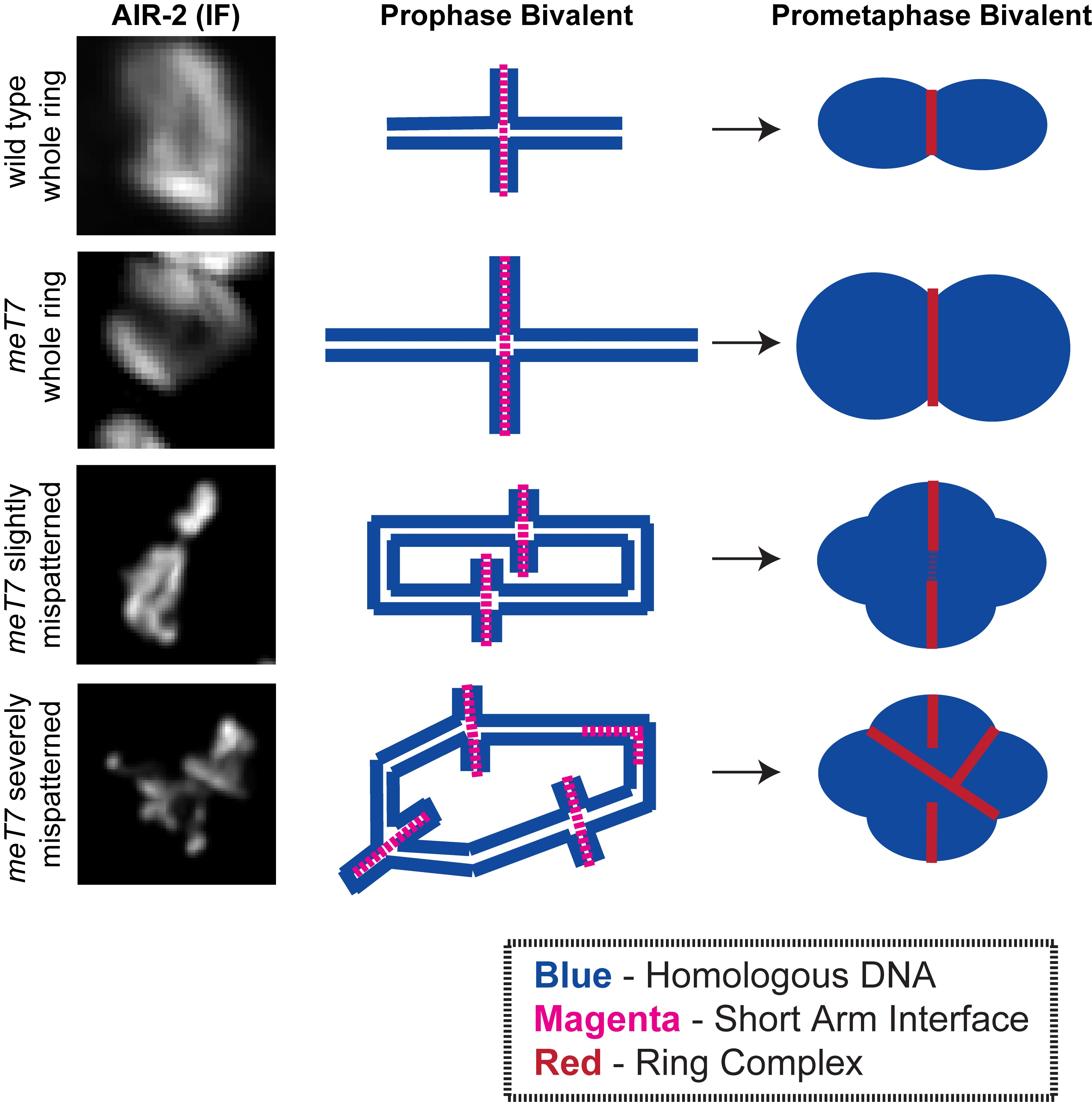

Supplement: S4 Fig — Cartoon images show potential models for how short arm interfaces are distributed on meT7 prometaphase bivalents, and how they may relate to different ring complex (RC) patterns from fixed oocyte zooms in Fig 2A. One whole ring (second row) may result from a single short arm interface, recruiting RC components in a manner similar to a wild-type-size bivalent (top row) on prometaphase bivalents. meT7 bivalents with a slight mispatterning RC (third row) may have two short arm interfaces, one at each end of the paired homologs, leading to a structure with ring complex components that are distinct, yet visible on the same plane. For bivalents with more complex RC mispatterning (bottom row), it is likely that the short arm interface is significantly impaired, potentially due to incorrect crossover number, and the targeted RC is not restricted to a single plane or a coherent, ordered structure in prometaphase. (TIF) [file pgen.1009001.s004.tif]

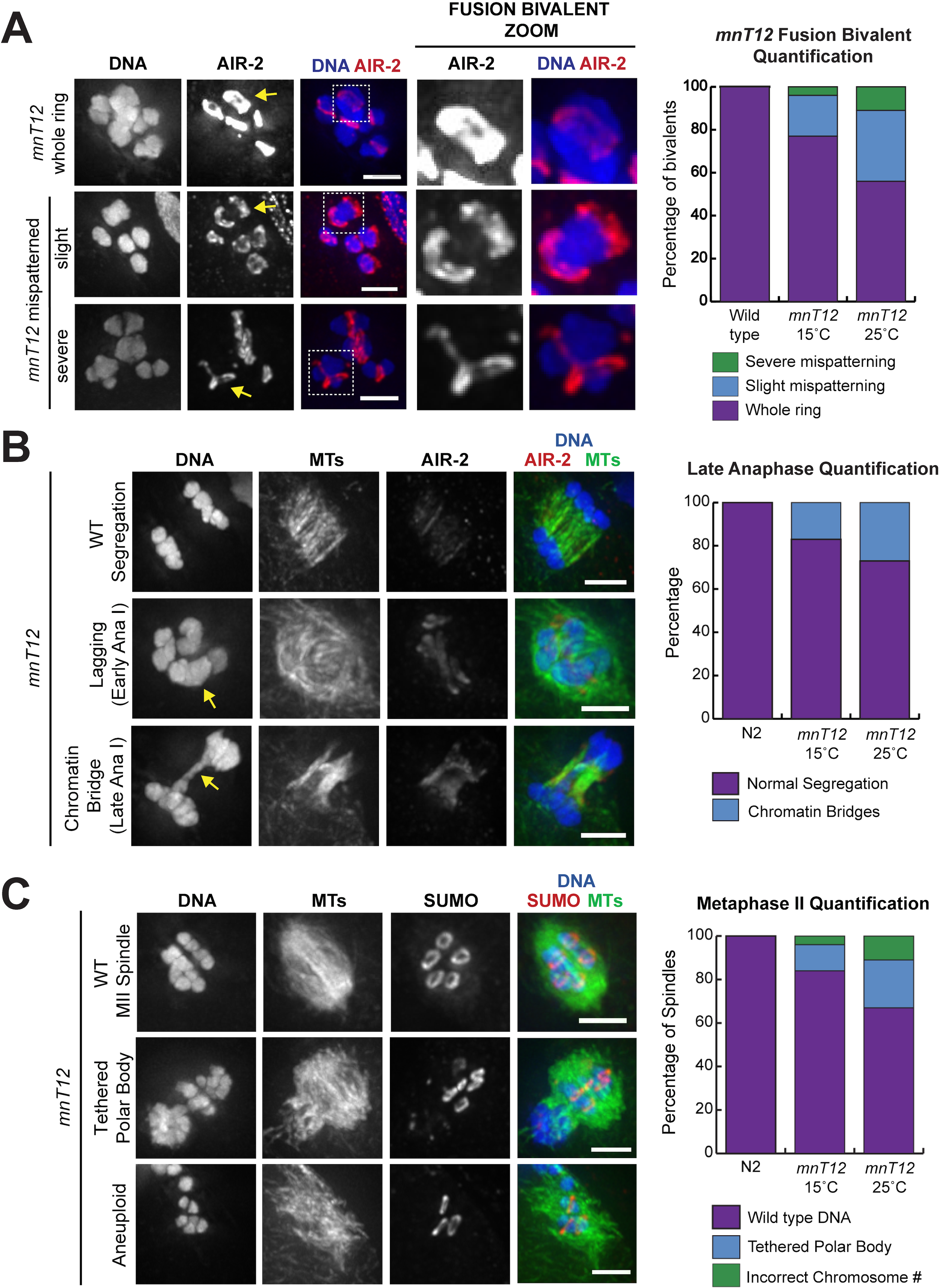

Supplement: S5 Fig — (A) Immunofluorescence and quantification of AIR-2 localization in fixed mnT12 oocytes. Single-bivalent zooms of mnT12, indicated by arrows, show that we see similar categories of AIR-2 localization as meT7: whole ring (top row), slight mispatterning (middle row), or severe mispatterning. 0% (0/40 at 15°C and 0/35 at 25°C) of normal bivalents showed mispatterned AIR-2 localization in mnT12 oocytes. At 15°C, mnT12 bivalents were slightly mispatterned in 8/36 oocytes and severely mispatterned in 1/36 oocytes, while at 25°C mnT12 bivalents were slightly mispatterned in 9/27 oocytes and severely mispatterned in 3/27 oocytes. (B) Chromatin bridges, indicated by arrows, are present in fixed mnT12 anaphase oocytes. In N2 mid-to-late anaphase oocytes, 0/75 spindles contained anaphase bridging. However, 3/18 mnT12 spindles showed chromatin bridges at 15°C, and 3/11 mnT12 anaphase spindles showed chromatin bridges at 25°C. (C) Fixed mnT12 oocytes show persisting consequences of anaphase bridging in Meiosis II. No chromatin-tethered polar bodies or anueploid Meiosis II spindles were observed in N2 Meiosis II oocytes. At 15°C, 3/25 Meiosis II mnT12 oocytes contained DNA tethered to the polar body, and 1/25 Meiosis II mnT12 oocytes was aneuploid. At 25°C, 6/27 Meiosis II mnT12 oocytes had tethered polar bodies, and 3/27 of Meiosis II mnT12 oocytes were aneuploid. All scale bars = 2.5μm. (TIF) [file pgen.1009001.s005.tif]

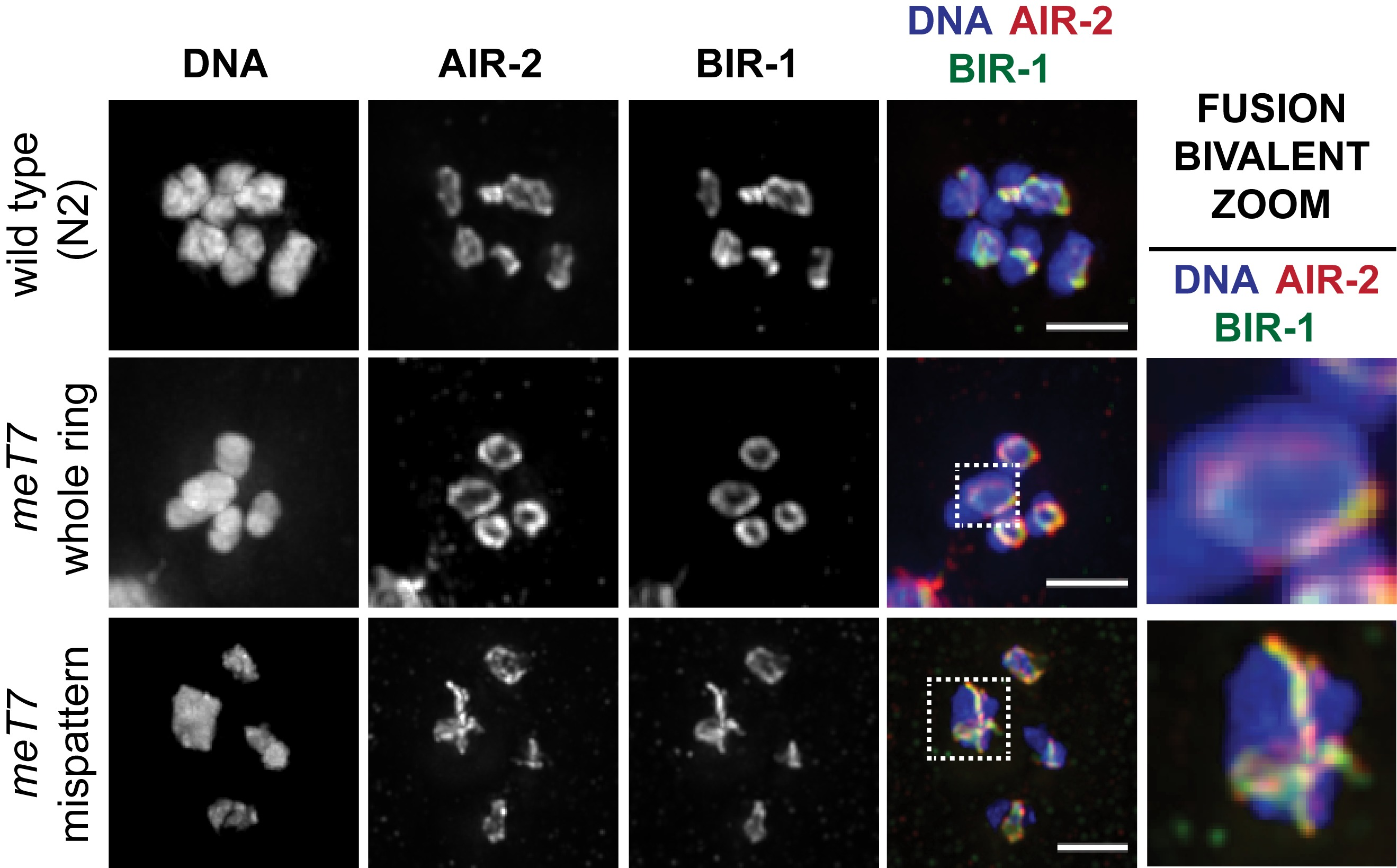

Supplement: S6 Fig — Immunofluorescence showing that BIR-1 (green) and AIR-2 (red) colocalize on all ring structure types on meT7 in fixed oocytes. Scale bars = 2.5μm. (TIF) [file pgen.1009001.s006.tif]

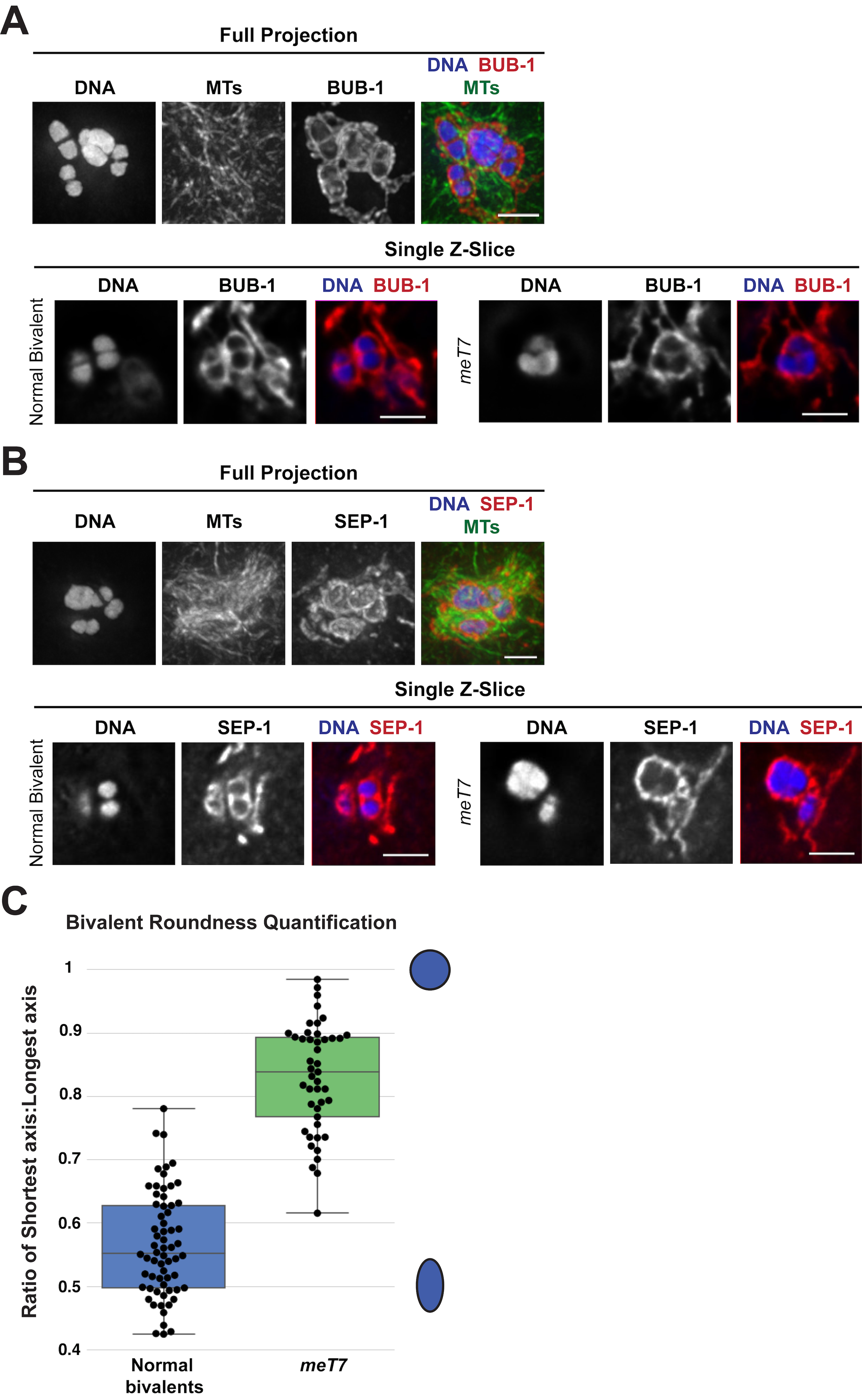

Supplement: S7 Fig — (A) Immunofluorescence of BUB-1 in prometaphase meT7 oocytes. BUB-1 targets to the entirety of meiotic bivalents in meT7, shown in a full projection (top row), and specifically cups holocentric normal bivalents (bottom row, left) and meT7 fused bivalents (bottom row, right), as shown in single slices. Scale bars = 2.5μm. (B) Immunofluorescence of SEP-1 in fixed prometaphase meT7 oocytes. SEP-1 targets to the entirety of meiotic bivalents in meT7, shown in a full projection (top row), and specifically cups holocentric normal bivalents (bottom row, left) and meT7 fused bivalents (bottom row, right), as shown in single slices. Scale bars = 2.5μm. (C) meT7 bivalents are shaped differently than normal-sized bivalents. Bivalent roundness, or the ratio of the length of the shortest axis on the bivalent to the length of the longest axis, was calculated using ImageJ. This ratio tended to be higher (P = 0.0027, two-tailed Mann-Whitney U test) for meT7 bivalents (N = 51) than normal-sized bivalents (N = 64), suggesting meT7 bivalents have a less apparent bilobed architecture, and instead have one closer to that of a circle. (TIF) [file pgen.1009001.s007.tif]
