# Supplementary material for: Excess crossovers impede faithful meiotic chromosome segregation in C. elegans
Source: PLoS Genet. 2020 Sep 4;16(9):e1009001. doi: 10.1371/journal.pgen.1009001 (PMC7508374; doi:10.1371/journal.pgen.1009001)
Supplement: S1 Table — (DOCX) [file pgen.1009001.s011.docx]

|  | **Condition** | | | |
| --- | --- | --- | --- | --- |
| **Phenotype** | **Wild Type** | **Wild Type**  ***lem-3(RNAi)*** | ***meT7 (III;X;IV)*** | ***meT7 (III;X;IV)* *lem-3(RNAi)*** |
| **Normal Segregation (MI and MII)** | 15/15 | 8/8 | 3/14 | 3/13 |
| **Normal Segregation MI, MII maybe lagging chromosome** | - | - | 2/14 | - |
| ***meT7* Bivalent Remains in Cytoplasm (MI)** | - | - | 1/14 | - |
| **MI Anaphase Bridge - Resolves** | - | - | 3/14 | - |
| **MI Anaphase Bridge – MII Spindle Tether** | - | - | 5/14 | 2/13 |
| **MI Anaphase Bridge – Possible Fragments** | - | - | - | 2/13 |
| **MI Fails Polar Body Extrusion** | - | - | - | 1/13 |
| ***meT7* Bivalent Extruded into Polar Body (MI)** | - | - | - | 5/13 |

**S1 Table. Frequency of chromosome segregation phenotypes from live imaging experiments.**
